# An annotated and illustrated checklist of Microgastrinae wasps (Hymenoptera, Braconidae) from the Canadian Arctic Archipelago and Greenland

**DOI:** 10.3897/zookeys.691.14491

**Published:** 2017-08-17

**Authors:** Jose Fernández-Triana, Joel Buffam, Melanie Beaudin, Hannah Davis, Ana Fernández-Galliano, Emily Griffin, Shang-Yao Lin, Megan K. McAulay, Robin Richter, Freddy Rodriguez, Gergely Várkonyi

**Affiliations:** 1 Canadian National Collection of Insects, 960 Carling Ave, Ottawa, K1A 0C6, Canada; 2 Institut für Biologie, Freie Universität Berlin, Königin-Luise-Str. 1-3, 14195 Berlin, Germany; 3 Ottawa, Ontario, Canada; 4 Department of Biology, University of Ottawa, 30 Marie Curie, Ottawa, K1N 6N5, Canada; 5 Finnish Environment Institute (SYKE), Natural Environment Centre, Lentiirantie 342 B, FI-88900 Kuhmo, Finland

**Keywords:** High Arctic, Microgastrinae, checklist, Citizen Science

## Abstract

The Microgastrinae (Hymenoptera: Braconidae) from ten islands of the Canadian Arctic Archipelago (CAA) and Greenland were studied based on 2,183 specimens deposited in collections. We report a total of 33 species in six genera, more than doubling the totals previously known. Most of the species (75.7%) have a distribution restricted to the Nearctic, with nine of those (27.3%) confirmed to be High Arctic endemics and another 10 species considered very likely to be High Arctic endemics as well – accounting for all of those, more than half of all species found are endemic to the region. The most diverse genera were *Cotesia* (10 species), *Glyptapanteles* (9 species), and *Microplitis* (7 species), representing 78.8% of the overall species diversity in the region. The six most frequently collected species comprised 84.7% of all examined specimens. The flight period for Microgastrinae in the High Arctic encompasses only two months, with activity peaking during the first half of July, when almost 40% of all available specimens were collected, and then plummeting in the first half to the end of August. Microgastrinae wasps from the High Arctic are currently known to parasitize eight species within four families of Lepidoptera: three species of Noctuidae, two each of Lymantridae and Nymphalidae, and one species of Pterophoridae. However, that information is very preliminary, as only six of the 33 species of microgastrines currently have associated host data. An annotated checklist, including photographs for 24 of the 33 species, is provided, as well as a key to all Microgastrinae genera present in the region.

## Introduction

The High Arctic land areas in North America comprise the Canadian Arctic Archipelago (CAA), with 36,500+ islands covering 1.42 million km^2^, and parts of Greenland, the world’s largest island with a total area of 2.17 million km^2^ (Danks 1981, Aiken et al. 2007, Böcher et al. 2015). High Arctic areas experience a very long and cold winter, with average temperatures of -25 to -35°C in the coldest months; while the summers are very short, with the average temperature during the warmest month (July) being less than 10°C (Aiken et al. 2007, Böcher et al. 2015).

By any of the geographically, climatically or botanically based definitions, the entire CAA and most of Greenland are unambiguously Arctic, lacking open forest or forest-tundra areas (Danks 1981, Aiken et al. 2007); only some inland areas along the fjords of Southwest and South Greenland have low birch forests and copse growth at protected sites (Böcher et al. 2015). The vascular plant diversity reaches 350 species in the CAA and almost 500 in Greenland (Danks 1981).

The insect fauna of the High Arctic areas in North America is rather poor in diversity. Approximately 360 species were reported by Danks (1981), about half of them being Diptera. A recent treatment of the Greenland ‘entomofauna’ (Böcher et al. 2015) significantly increased the total for that island to around 1,200 reported species, but that figure included non-insect groups such as Collembola, Arachnida and Chilopoda (the insect diversity recorded in that work was around 800 species, with the actual figure not clear as a relatively large number of species were recorded as ‘likely’ but not ‘confirmed’ for Greenland).

Parasitoid wasps (Hymenoptera), one of the most conspicuous and diverse animal groups on Earth (LaSalle and Gauld 1991, 1993, Quicke 1997), comprise the second most diverse group of insects in the High Arctic after Diptera. At least 80 species were reported by Danks (1981: 199), but that total has already increased significantly, as there are now almost 200 species of parasitoid wasps known from Greenland alone (Böcher et al. 2015), and other works have been published on the CAA (e.g., Timms et al. 2013). There is no question that more studies on the High Arctic fauna will further increase that figure.

With 2,710 described species worldwide and several thousand more undescribed (Rodriguez et al. 2013, Yu et al. 2016), the subfamily Microgastrinae (Braconidae) is the single most important group of parasitoid wasps attacking caterpillars (Lepidoptera) (Whitfield 1997). Two genera and six unnamed species of Microgastrinae were recorded from the High Arctic areas by Danks (1981: 200, 514–515), but recent studies have increased those figures. A total of 13 species within three genera have been reported from Greenland (van Achterberg 2006, 2015), and Fernández-Triana et al. (2009) and Fernández-Triana (2010) estimated 20 to 25 species to be present in Canada at the 70–80°N latitudinal range (which includes the CAA but also other areas from mainland North America), although he did not provide any specific details on the identity of those species.

Here we update the information on the Microgastrinae fauna of the CAA and Greenland, including an annotated and illustrated checklist of species, as well as a key to all Microgastrinae genera present in the region. Additionally, this paper presents the first results of a Citizen Science project initiated by the Canadian National Collection of Insects (CNC), as part of the Ottawa 2016 Bug Day (http://www.entsocont.ca/bug-day-ottawa-2016.html), as specimen databasing and pictures were mostly done by volunteers.

## Methods

For this paper we follow the traditional definition of the CAA that is detailed in other sources (e.g., Danks 1981, Aiken et al. 2007). From east to west, the CAA extends from the eastern tip of Baffin Island (61°15'W) to the southwest corner of Banks Island (125°49'W), a distance of about 3,000 km. In a north-south direction, it extends from Cape Columbia on the north coast of Ellesmere Island (83°39'N) to Akpatok Island (60°12'N), which is also a distance of about 3,000 km. Greenland lies between latitudes 59° and 83°N, and longitudes 11° and 74°W.

**Figure 1.**
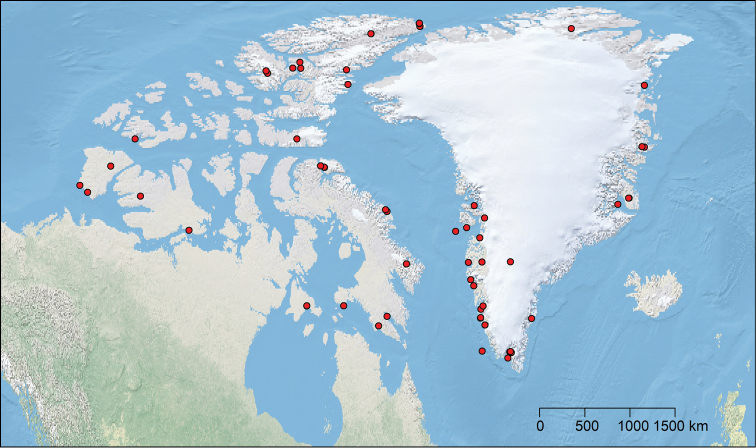
Greenland and Canadian Arctic Archipelago localities included in the present study.

We studied all specimens deposited in the CNC, as well as 25 specimens from the Biodiversity Institute of Ontario collection. We also incorporated information from specimens mentioned in previous papers (van Achterberg 2006, 2015, Várkonyi and Roslin 2013), which are deposited in collections in Denmark, Finland and Hungary. In total 2,183 specimens are included in this paper from Greenland and the following 10 islands of the CAA: Axel Heiberg, Baffin, Banks, Bylot, Devon, Dorset, Ellesmere, Melville, Southampton and Victoria. Localities studied are shown in Figure 1, and in Suppl. material 1. The map was generated using R (the code used to provide the map is provided in Suppl. material 1).

For consistency, localities from mainland North America were excluded, despite the fact that some of them are at higher latitudes than some of the CAA islands covered in this paper (e.g., Boothia and Melville Peninsulas).

Specimens were identified and assigned to species following the most recent taxonomic information available for the region (Fernández-Triana 2010, Fernández-Triana et al. 2011, van Achterberg 2006, 2015). Some specimens could only be identified to genus and were given an alphanumeric species identifier, e.g., ‘*Cotesia* sp. 1’: in all such cases, unique morphological characteristics and/or DNA barcodes clearly identified them as distinct species. In order to allow these provisional species to be recognized and studied further in the future, we also provide DNA Barcodes Index Numbers (BINs) (Ratnasingham and Hebert 2013) for them in the annotated species checklist.

Pictures of 24 species are provided to illustrate the diversity of microgastrine wasps in the High Arctic. Photos were taken with a Keyence VHX-1000 Digital Microscope, using a lens with a range of 10–130 ×. Multiple images were taken of a structure through the focal plane and then combined to produce a single in-focus image using the software associated with the Keyence System. Plates were prepared using Microsoft PowerPoint 2010.

A key to all genera of Microgastrinae present in CAA and Greenland is provided. Morphological terms follow Mason (1981), Huber and Sharkey (1993), Whitfield (1997), Karlsson and Ronquist (2012), and Fernandez-Triana et al. (2014).

A species checklist was generated using the CNC database (http://www.cnc-ottawa.ca/taxonomy/TaxonMain.php). The list is organized alphabetically by genus and species within a given genus. For every taxon we detail general distribution (outside of the High Arctic), specimens examined, and notes on species where relevant. For zoogeographic regions we use the following acronyms: NEA-Nearctic, OTL-Oriental, and PAL-Palearctic. The acronym BOLD refers to Barcode of Life Data Systems (http://v4.boldsystems.org/index.php).

A Citizen Science project to database parasitoid wasp specimens deposited in the CNC started during the Ottawa 2016 Bug Day (http://www.entsocont.ca/bug-day-ottawa-2016.html). As part of that project, volunteers photographed specimen labels and later transcribed them into the CNC database. Some of the species photographs used in this paper were also taken by participants in that project.

## Results and discussion

At least 33 species within six genera of Microgastrinae were found in the High Arctic (Table 1), more than double the totals previously published (around 12 species and three genera, Oliver 1963, Danks 1981, van Achterberg 2006, 2015, Várkonyi and Roslin 2013, Böcher et al. 2015, Wirta et al. 2016). We also found a few additional species, but were unable to include them in the present paper as the available specimens were in poor condition or could not be studied (we list their voucher codes at the end of the checklist). Investigation of more material from other islands and/or additional specimens from other collections will likely increase the total diversity of this group of parasitoid wasps for the region.

The diversity of Microgastrinae in the High Arctic, as revealed in this paper, can be considered extraordinary. It had previously been estimated that very few species of Hymenoptera were present in that region, but our results show that the number of species is much higher than previously anticipated. For example, Danks (1981: 200, 514–515) estimated that only six species of Microgastrinae occur in the High Arctic. Just from the northern tip of Ellesmere Island, in the localities of Alert (82.5°N) and Hazen Camp (81.8°N) we report here for the first time a total of five species within four different genera of Microgastrinae: *Cotesia eliniae* Papp, 1989, *C. hallii* (Packard, 1877), *Dolichogenidea sicaria* (Marshall, 1885), *Glyptapanteles* sp. 5 and *Microplitis coactus* (Lundbeck, 1896).

Even in the more studied areas, the increase in the number of species and genera of Microgastrinae is still significant. Achterberg (2006) recorded 14 species within three genera for Greenland, but later revised that total down to 12 species (van Achterberg 2015); while Várkonyi and Roslin (2013) added one genus (*Dolichogenidea*) but did not specify the species. Here we add five species and record one additional genus to the fauna of Greenland, which represents the highest diversity of all islands studied with 17 species in five genera (Table 1). Those figures are no doubt the result of Greenland being the largest island, and also the one most intensively sampled (more than half of all specimens considered for this paper) and studied for the longest period of time, as well as having the most diverse vegetation, including subarctic elements (Danks 1981, Böcher et al. 2015).

There were no previous records of Microgastrinae species published for the CAA. Oliver (1963) and Danks (1981) mentioned “*Apanteles* spp.” and “*Microplitis* spp.” as two genera present in the CAA without further details (but the genus *Apanteles* has not actually been found so far in the High Arctic, see below). In addition, Fernández-Triana (2010) mentioned that the genus *Glyptapanteles* in Canada reached the tip of Ellesmere Island (+82°N), but did not elaborate this further. Here we record 26 species of Microgastrinae for ten islands of the CAA (Table 1), with Banks (11 species), Baffin (10), Ellesmere (9) and Victoria (7) islands harbouring the highest diversity. These totals are also likely correlated to the collecting effort done, which is far from being uniform between the studied islands.

**Table 1.** High Arctic species of Microgastrinae (Hymenoptera, Braconidae), their distribution per island and associated host information (when known). Legend: * - Indicates a new species record for a specific island. ** - Indicates a new host record for the wasp species. (1) - Based on published information only, the species would be considered a High Arctic endemic; however, unpublished data in the BOLD database reveals that the species is also found on mainland North America south of the High Arctic (and thus it is not counted as an endemic in the final row ‘TOTAL’) . (?)- Probable High Arctic endemic species. X(?)- Indicates a dubious species record. 9(10)- Nine species are currently reported to be High Arctic endemics, with another 10 species considered as potential endemics.

	High Arctic Endemic	Green land	Axel Heinberg	Baffin	Banks	Bylot	Devon	Dorset	Ellesmere	Melville	Southampton	Victoria	Host information
*Cotesia crassifemorata* van Achterberg, 2006	**X**	**X**											Unknown
*Cotesia eliniae* Papp, 1989	**X**	**X**	X*		X*		X*		X*	X*		X*	Unknown
*Cotesia fascifemorata* van Achterberg, 2006	**X**	**X**											Unknown
*Cotesia hallii* (Packard, 1877)	**(1)**	**X**	X*	X*	X*	X*	X*		X*	X*	X*	X*	Lymantridae: *Gynaephora groenlandica* **
*Cotesia yakutatensis* (Ashmead, 1902)		**X**		X*									Noctuidae (in areas south of the High Arctic)
*Cotesia* sp. 1	**X**										X*		Lymantridae: *Gynaephora* sp.**
*Cotesia* sp. 2	**(1)**					X*							Unknown
*Cotesia* sp. 3	**X**				X*								Unknown
*Cotesia* sp. 4	**X**				X*								Unknown
*Cotesia* sp. 5	**X**	X*			X*				X*				Unknown
*Dolichogenidea sicaria* (Marshall, 1885)		X*	X*	X*					X*				Pterophoridae: *Stenoptilia islandica* (potential host record from Greenlandic specimens (Várkonyi and Roslin 2013)). Eleven families and 33 species known as hosts in areas south of the High Arctic
*Dolichogenidea* sp. 1	**(?)**			X*	X*								Unknown
*Dolichogenidea* sp. 2	**(?)**				X*								Unknown
*Dolichogenidea* sp. 3	**(?)**											X*	Unknown
*Glyptapanteles compressiventris* (Muesebeck, 1921)				X*				X*					Three families (mainly Arctiidae) and 10 species in areas south of the High Arctic
*Glyptapanteles fulvipes* (Haliday, 1834)		**X**	X*	X*					X*			X*	Numerous families and species of Lepidoptera in areas south of the High Arctic (some of those records are dubious)
*Glyptapanteles pallipes* (Reinhard, 1880)		**X**											Numerous families and species of Lepidoptera in areas south of the High Arctic (some of those records are dubious)
*Glyptapanteles* sp. 1	**(?)**			X*	X*	X*							Unknown
*Glyptapanteles* sp. 2	**(1)**	X*		X*									Unknown
*Glyptapanteles* sp. 3	**(?)**				X*								Unknown
*Glyptapanteles* sp. 4	**(?)**			X*								X*	Unknown
*Glyptapanteles* sp. 5	**X**	X*							X*				Noctuidae: *Polia richardsoni* **
*Glyptapanteles* sp. 6	**(?)**								X*				Unknown
*Illidops* sp. 1	**(?)**	X*											Unknown
*Illidops* sp. 2	**(?)**			X*			X*			X*		X*	Unknown
*Microgaster* sp.					X*								Unknown
*Microplitis coactus* (Lundbeck, 1896)		**X**					X*		X*				Noctuidae: *Noctua* sp.
*Microplitis lugubris* (Ruthe, 1860)		**X**							X*				Noctuidae: *Sympistis nigrita*. Two other species (Noctuidae and Erebidae) are also recorded in areas south of the High Arctic
Microplitis sp. nr. lugubris	**(1)**					X*							Unknown
*Microplitis lugubroides* van Achterberg, 2006	**X**	**X**											Unknown
*Microplitis mandibularis* (Thomson, 1895)		**X**											Noctuidae: several species (in areas south of the High Arctic)
*Microplitis sofron* Nixon, 1970		**X(?)**											Noctuidae: *Tholera cespitis* (in areas south of the High Arctic)
Microplitis sp. nr. sofron	**(?)**				X*							X*	Unknown
**TOTAL**	**9 (10)**	**17**	**4**	**10**	**11**	**4**	**4**	**1**	**9**	**3**	**2**	**7**	

Most of the species (25, representing 75.7% of the total) had a distribution restricted to the Nearctic, while seven species (21.2%) had a Holarctic distribution (Nearctic and Palaearctic), and only one species had a wider distribution (Nearctic, Palaearctic and Oriental). Nine species (27.3%) are confirmed in this paper to be High Arctic endemics. Another 10 species are very likely to be High Arctic endemics as well – if accounting for all of those, then more than half of all species found in Greenland and the CAA are endemic to the region.

The most diverse genera were *Cotesia* (10 species), *Glyptapanteles* (9 species), and *Microplitis* (7 species). Those three genera accounted for 78.8% of the overall species diversity in the region. *Apanteles*, currently the most diverse and widespread genus of Microgastrinae with over 1,000 species worldwide (Yu et al. 2016), was notably absent from the High Arctic samples we could examine. However, that genus is present in mainland North America (in localities of similar latitude and habitats than the CAA), so it is likely that the genus will eventually be found in the High Arctic when more studies are done and additional samples from the region are analyzed.

The most frequently collected species were *Microplitis lugubris* (Ruthe, 1860) (716 specimens), *Cotesia hallii* (575 specimens), *Glyptapanteles fulvipes* (Haliday, 1834) (243 specimens), *Cotesia eliniae* (129 specimens) *Microplitis coactus* (117 specimens), and *Dolichogenidea* sp. 1 (105 specimens). Those six species altogether accounted for 87.4% of all High Arctic specimens examined by us.

In contrast to Greenland, where research activity has been rather high recently (e.g., van Achterberg 2006, 2015, Várkonyi and Roslin 2013, Roslin et al. 2013, Wirta et al. 2014, Wirta et al. 2016), most of the specimens from the CAA were collected during early to mid 20^th^ century (Table 2). That is likely a reflection of the funding opportunities for Arctic research at the time, e.g., the Northern Insect Survey (Freman 1952, Freeman and Twinn 1955, Buddle et al. 2008, Fernández-Triana et al. 2009).

**Table 2.** Microgastrinae specimens collected in the Canadian Arctic Archipelago during successive time periods between 1930–2014. Data from present paper.

Time period	1930–1959	1960–1989	1990–2014
Specimens collected (%)	28.6	55.5	15.8

**Figure 2.**
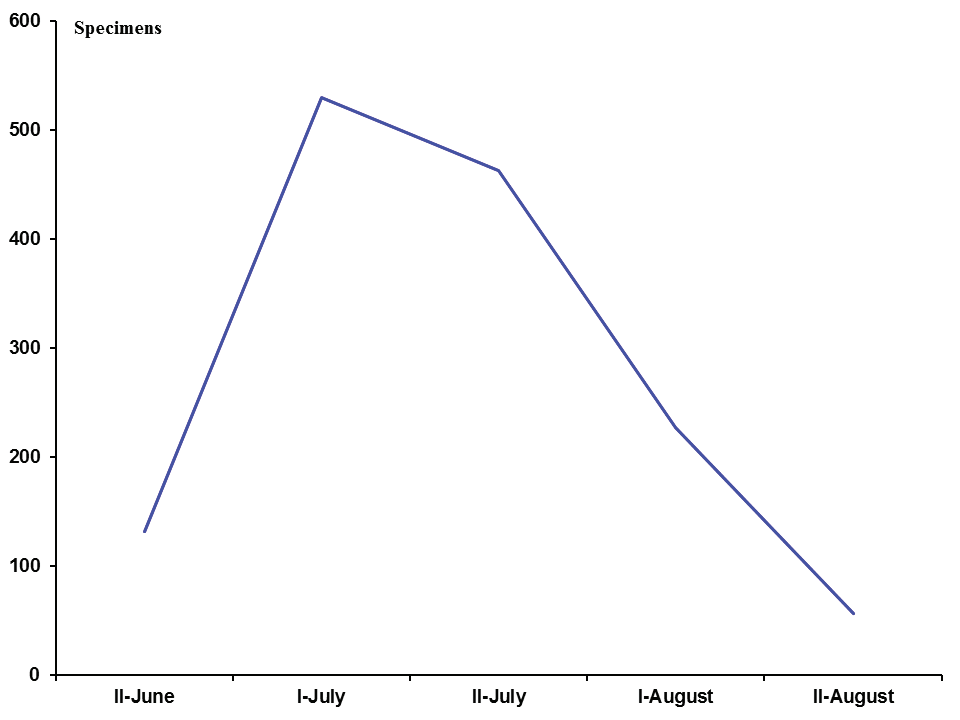
Flight period of Microgastrinae in the High Arctic. Number of specimens (as shown on Y axis) based on data from present paper. **I** First half of a month **II** Second half of a month.

Based on the studied specimens, the flight period for Microgastrinae in the High Arctic encompasses only two months, from the second half of June to the second half of August. There were less than 20 specimens with collecting dates of late May/early June or early September, but all came from the southernmost localities in the studied region. Activity peaks during the first half of July, when almost 40% of all available specimens were collected. The number of specimens drops slightly during the second half of July and then plummets in the first half to end of August, marking the end of the flying season for Microgastrinae in the region (Figure 2). This is by far the shortest flying season we have observed for microgastrine wasps worldwide, although it is otherwise expected due to the high latitude and very low temperatures in the High Arctic.

The majority (82%) of the High Arctic species of Microgastrinae have no host data available. Only six of the 33 species analyzed in this paper have some Lepidoptera recorded as hosts, with three of those species being new records reported here: *Cotesia hallii* parasitizing *Gynaephora groenlandica* (Lymantridae), *Cotesia* sp. 1 as a parasitoid of *Gynaephora* sp. (Lymantridae), and *Glyptapanteles* sp. 5 parasitizing *Polia richardsoni* (Noctuidae).

There are two additional host records for unnamed species of Microgastrinae in the High Arctic. Várkonyi and Roslin (2013) reported “*Dolichogenidea* sp.” as a parasitoid of *Stenoptilia islandica* (Pterophoridae) in Greenland. *Dolichogenidea sicaria* is the only species of that genus known from Greenland and the specimens from Várkonyi & Roslin (2013) have DNA barcodes that clearly match (see comments under that species in the Checklist below and also the Supplementary Info file in Wirta et al. (2016)), thus we consider here that the “*Dolichogenidea* sp.” specimens mentioned by Várkonyi & Roslin (2013) and Wirta et al. (2016) belong to *D. sicaria*.

Várkonyi and Roslin (2013) also reported “*Cotesia* spp.” as important parasitoids of *Boloria chariclea* and *B. polaris* (Nymphalidae) in Greenland. According to those authors, the identity of their “*Cotesia* spp.” specimens is more difficult to establish, but it is likely to be in the *C. eliniae* / *C. hallii* species complex. Unfortunately at this point we cannot conclude with certainty about the species identity of those *Cotesia* specimens.

When including all the information available, the Microgastrinae from the High Arctic are currently known to attack eight species within four families of Lepidoptera: three species of Noctuidae, two each of Lymantridae and Nymphalidae, and one species of Pterophoridae (Table 1).

### Key to genera of Microgastrinae found in the Canadian Arctic Archipelago and Greenland

**Table:** 

1	Fore wing with areolet (second submarginal cell entirely closed by veins)	**2**
–	Fore wing wihout areolet (second submarginal cell not entirely closed by veins)	**3**
2(1)	Posterior margin of anteromesoscutum with sharply defined carina right before scuto-scutellar sulcus; scutellar disc with band of rugosity centrally on posterior margin; mediotergite 1 relatively long and narrow (length centrally 2.0 × or more its width at posterior margin), not widening towards posterior margin in High Arctic species; mediotergite 2 mostly smooth and poorly defined laterally; metacoxa relatively small, not surpassing posterior margin of mediotergite 2; metatibial spurs less than half length of first segment of metatarsus	***Microplitis***
–	Posterior margin of anteromesoscutum without carina right before scuto-scutellar sulcus; scutellar disc without band of rugosity centrally on posterior margin; mediotergite 1 relatively broad (length centrally 1.0 × or less its width at posterior margin), strongly widening towards posterior margin; mediotergite 2 heavily sculptured and rectangular-shaped; metacoxa relatively large, surpassing posterior margin of mediotergite 2; metatibial spurs more than half length of first segment of metatarsus	***Microgaster***
3(2)	Ovipositor sheaths relatively short, its length less than half metatibia length, usually much shorter	**4**
–	Ovipositor sheaths relatively long, its length close to or longer than metatibia length	**5**
4(3)	Propodeum heavily sculptured, with median carina and usually partial to complete transverse carinae (which might be obscured by strong sculpture on entire propodeum); mediotergite 1 heavily sculptured and relatively broad (length centrally 1.0 × or less its width at posterior margin), strongly widening towards posterior margin; mediotergite 2 heavily sculptured and rectangular-shaped	***Cotesia***
–	Propodeum slightly sculptured or entirely smooth, median carina rarely complete (usually only defined partially on posterior half, sometimes entirely absent), transverse carinae always absent; mediotergite 1 mostly to entirely smooth, relatively long and narrow (length centrally 2.0 × or more its width at posterior margin), narrowing towards posterior margin; mediotergite 2 mostly to entirely smooth, subtriangular to trapezoidal in shape	***Glyptapanteles***
5(3)	Scutellar disc with band of rugosity centrally on posterior margin; propodeum heavily sculptured but without defined areola; fore wing with vein R1 shorter than pterostigma length; head in frontal view with eyes converging ventrally	***Illidops***
–	Scutellar disc without band of rugosity centrally on the posterior margin; propodeum slightly sculptured to smooth, with partial to completely defined areola; fore wing with vein R1 longer than pterostigma length; head in frontal view with eyes not converging ventrally	***Dolichogenidea***

### Checklist of species

#### Cotesia crassifemorata

Taxon classificationAnimaliaHymenopteraBraconidae

van Achterberg, 2006

##### Distribution.

NEA. High Arctic endemic.

##### Notes.

Only known from the original description; from Greenland (van Achterberg 2006).

#### Cotesia eliniae

Taxon classificationAnimaliaHymenopteraBraconidae

Papp, 1989

Fig. 3

##### Distribution.

NEA. High Arctic endemic (the species was described from Scoresby Sund, which is at the northern boundary of Low Arctic zone).

**Figure 3.**
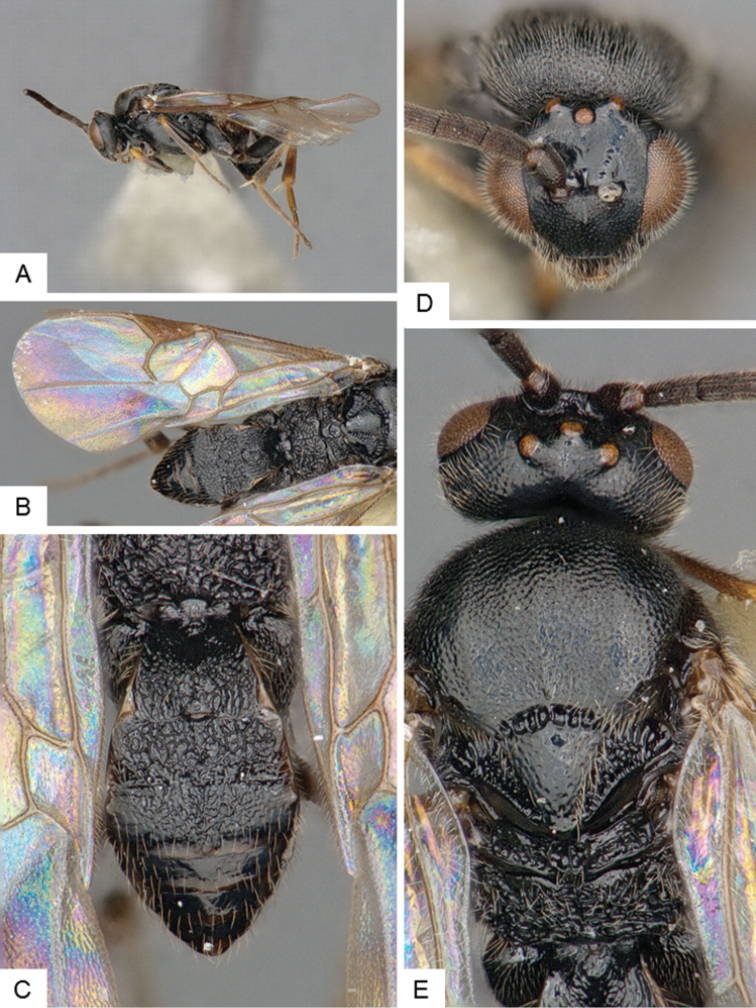
*Cotesia eliniae*. **A** Habitus, lateral **B** Fore wing **C** Metasoma, dorsal **D** Head, frontal-dorsal **E** Head and mesosoma, dorsal.

##### Notes.

Previously only known from Greenland, here also recorded from the CAA islands of Axel Heiberg, Banks, Devon, Ellesmere, Melville, and Victoria. The DNA barcodes of a few specimens cluster with some sequences of *C. hallii* and it is not clear if these two are indeed different species. The keys provided by van Achterberg (2006, 2015), based on Greenland material, do not always work for CAA specimens –nor for other Greenlandic specimens (e.g., Várkonyi and Roslin (2013)). The only reliable character that seems to delineate species is the sculpture on mediotergite 3, which ranges from almost to fully sculptured in *C. eliniae*, whereas is almost to fully smooth in *C. hallii*. No host record is known for *C. eliniae*. One series of specimens from Ellesmere, collected by J.R. Smith on July 1980, were reared from an unspecified caterpillar (the labels have no information on the identity of the host, but just a code number: ‘217’). The available DNA sequences for this species correspond in BOLD to BIN BOLD:ACE6464.

#### Cotesia fascifemorata

Taxon classificationAnimaliaHymenopteraBraconidae

van Achterberg, 2006

##### Distribution.

NEA. High Arctic endemic.

##### Notes.

Only known from the original description; from Greenland (van Achterberg 2006).

#### Cotesia hallii

Taxon classificationAnimaliaHymenopteraBraconidae

(Packard, 1877)

Fig. 4

##### Distribution.

NEA. High Arctic and some additional, unpublished records in BOLD from northern Canada (mainland).

**Figure 4.**
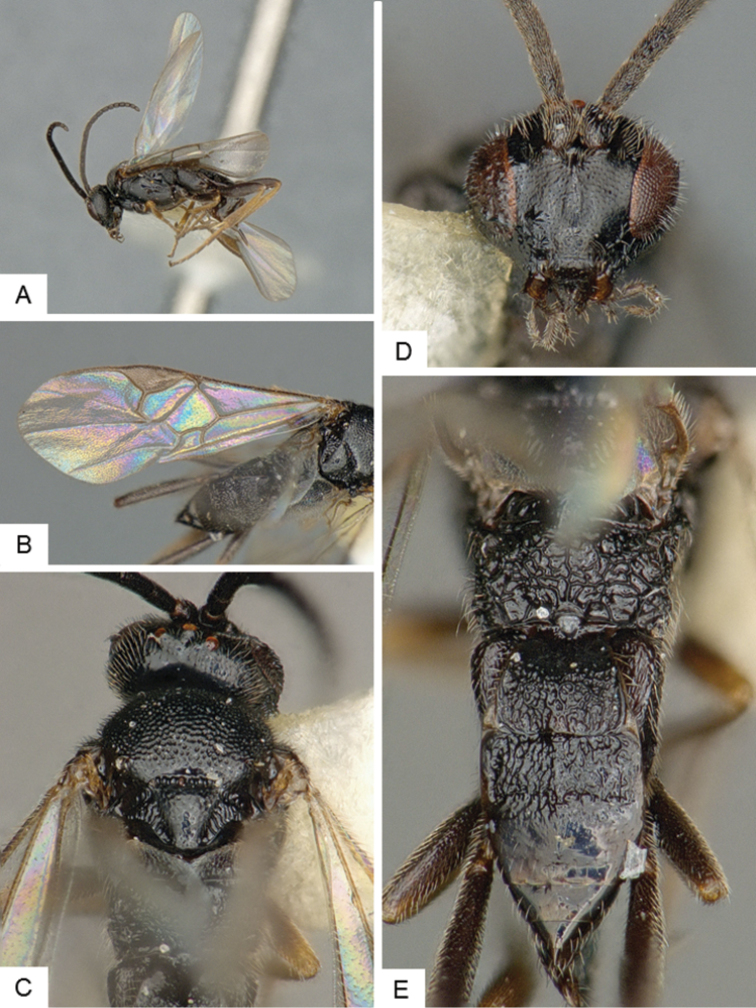
*Cotesia hallii*. **A** Habitus, lateral **B** Fore wing **C** Head and mesosoma, dorsal **D** Head, frontal **E** Metasoma, dorsal.

##### Notes.

A total of 575 specimens from Greenland and nine islands in the CAA: Axel Heiberg, Baffin, Banks, Bylot, Devon, Ellesmere, Melville, Southampton, and Victoria. Host: *Gynaephora groenlandica* (Wocke, 1874) (Lymantridae), records based on two wasp specimens (with voucher codes MIC 000317, MIC 000320) from Eureka, Ellesmere Island, and a series of 24 specimens (voucher codes CNC492946- CNC492969) from Devon Island. They represent the first known record of a Braconidae parasitizing *G. groenlandica*. The available DNA sequences for this species correspond in BOLD to BIN BOLD:AAA5700.

#### Cotesia yakutatensis

Taxon classificationAnimaliaHymenopteraBraconidae

(Ashmead, 1902)

Fig. 7

##### Distribution.

NEA.

##### Notes.

This species is rather widely distributed in the Nearctic. It had previously been recorded from Greenland by Papp (1989), and here we also recorded it from the CAA for the first time, based on two specimens collected in Baffin and Bylot Islands (voucher codes CNC497416, CNCH0395). It is clearly a southern species, scarcely reaching its northernmost range in the High Arctic. The majority of the specimens identified in BOLD as *C. yakutatensis* correspond to BIN BOLD:ABZ4485, but other specimens, including the one from Bylot Island, actually belong to BIN BOLD:AAA5701. Solving the limits of *C. yakutatensis* will require examination of specimens from across the species range, which is beyond the scope of the present paper.

#### Cotesia

Taxon classificationAnimaliaHymenopteraBraconidae

sp. 1

##### Distribution.

NEA. High Arctic endemic.

##### Notes.

A series from Southampton Island (five specimens mounted but more than 40 additional specimens in alcohol, kept with the host remains and wasp cocoons mass). The species is morphologically similar to *C. hallii* and *C. eliniae*, but is distinctive because of the very strong and deep sculpture on mediotergites 1, 2 and at least anterior 0.2–0.3 of mediotergite 3 (in contrast, *C. eliniae* has most of mediotergites 1–3 sculptured, but the sculpture is much finer and mat). One of the specimens (voucher code CAM0668) has a partial barcode (421 base pairs) which is unique among all *Cotesia* specimens in BOLD, and rather different from those of *C. hallii* and *C. eliniae*. Host: *Gynaephora* sp. (Lymantridae).

#### Cotesia

Taxon classificationAnimaliaHymenopteraBraconidae

sp. 2

Fig. 5

##### Distribution.

NEA. High Arctic and some additional, unpublished records in BOLD from northern Canada (mainland).

**Figure 5.**
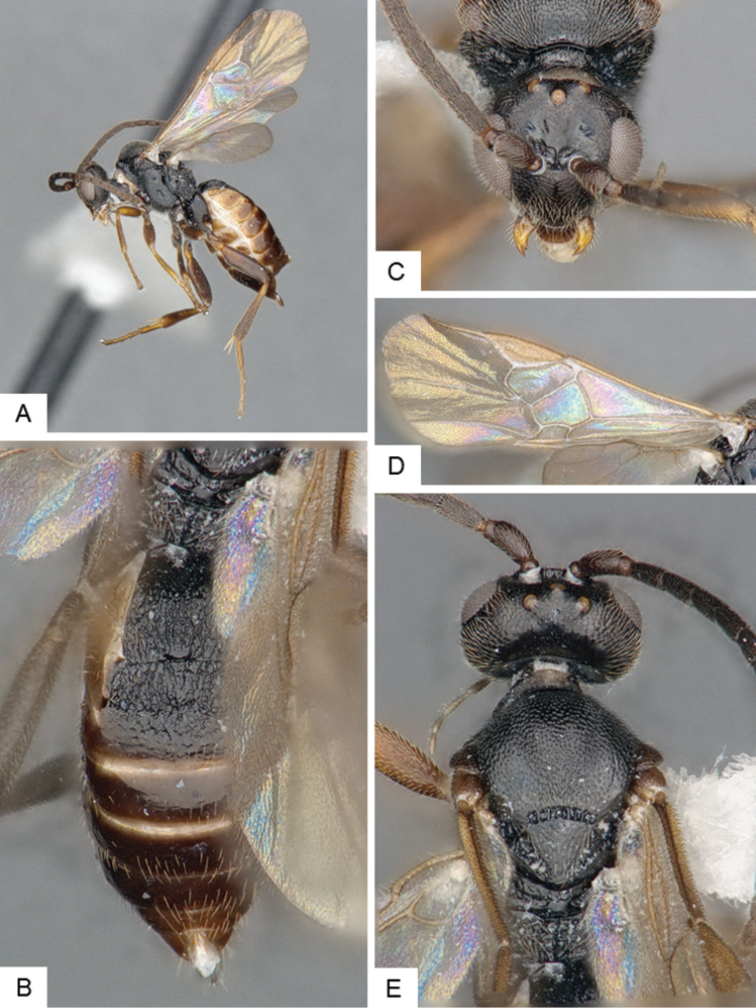
*Cotesia* sp. 2. **A** Habitus, lateral **B** Metasoma, dorsal **C** Head, frontal-dorsal **D** Fore wing **E** Head and mesosoma, dorsal.

##### Notes.

One female specimen from Bylot Island (voucher code CAM 0574). It has a large hypopygium, which extends beyond the end of the tergites. We have seen additional specimens from this species in Naujaat (known until 2015 as Repulse Bay), a locality in mainland Nunavut, Canada.

#### Cotesia

Taxon classificationAnimaliaHymenopteraBraconidae

sp. 3

Fig. 6

##### Distribution.

NEA. High Arctic endemic.

**Figure 6.**
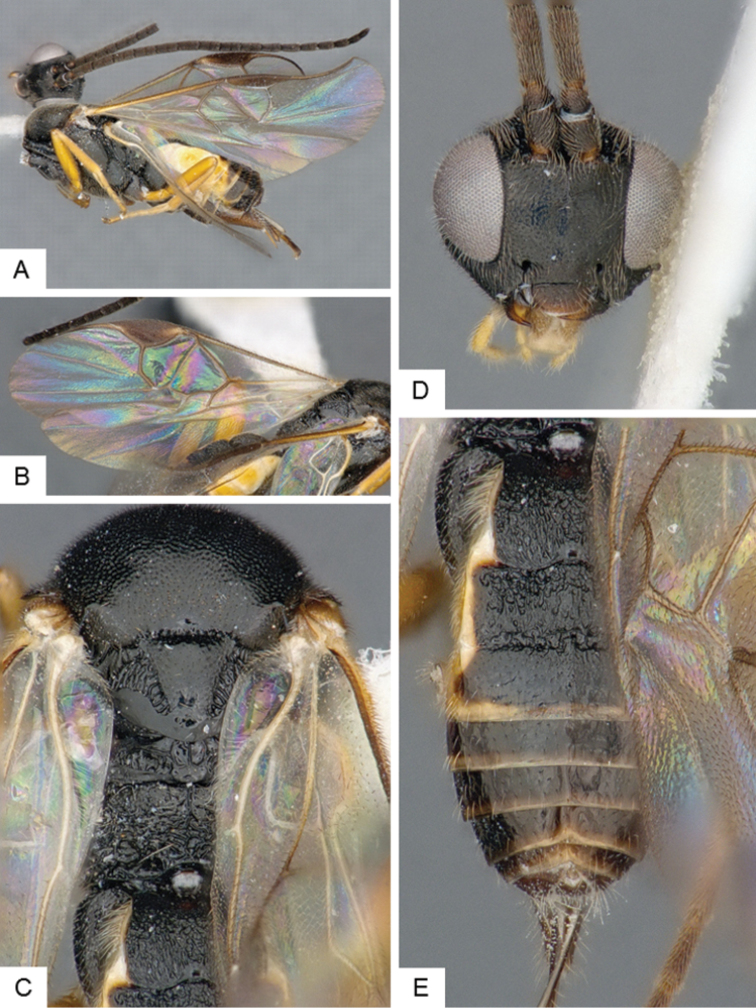
*Cotesia* sp. 3. **A** Habitus, lateral **B** Fore wing **C** Mesosoma, dorsal **D** Head, frontal **E** Metasoma, dorsal.

##### Notes.

One female specimen from Banks Island (voucher code GOU 0520). It has a unique DNA barcode and morphology. The available DNA sequences for this species correspond in BOLD to BIN BOLD:AAI6054.

**Figure 7.**
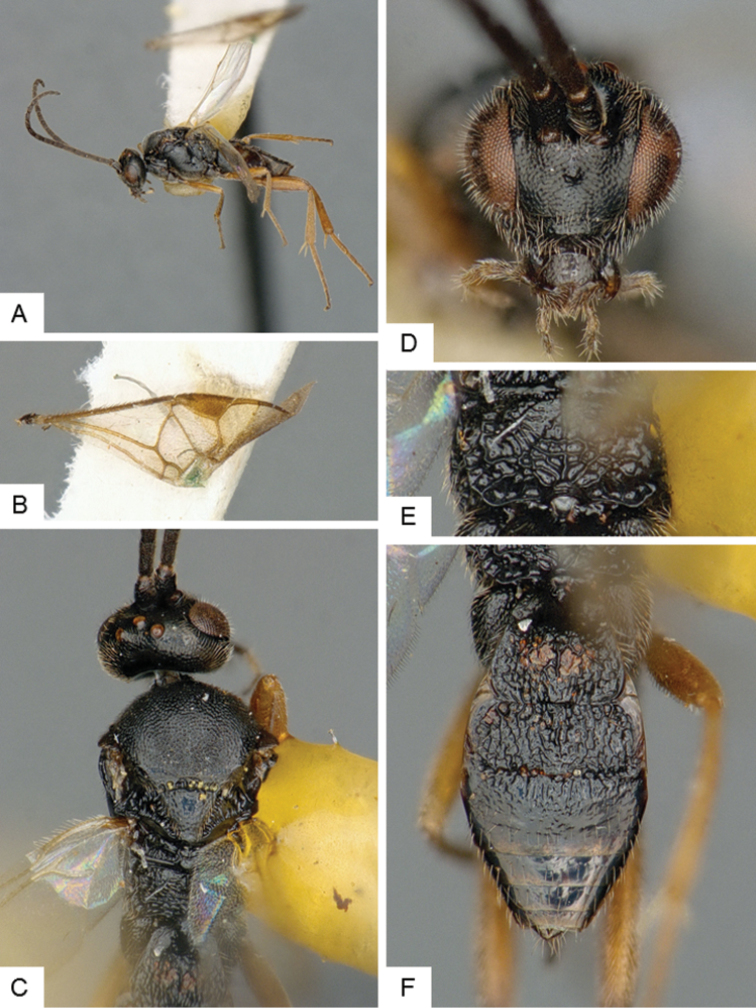
*Cotesia yakutatensis*. **A** Habitus, lateral **B** Fore wing **C** Head and mesosoma, dorsal **D** Head, frontal **E** Propodeum **F** Metasoma, dorsal.

#### Cotesia

Taxon classificationAnimaliaHymenopteraBraconidae

sp. 4

##### Distribution.

NEA. High Arctic endemic.

##### Notes.

One male specimen from Banks Island (voucher code GOU 0524). It has a unique DNA barcode and morphology. The available DNA sequences for this species correspond in BOLD to BIN BOLD:ACE3031.

#### Cotesia

Taxon classificationAnimaliaHymenopteraBraconidae

sp. 5

##### Distribution.

NEA. High Arctic endemic.

##### Notes.

A total of 16 specimens from Banks and Ellesmere Islands, as well as Greenland. This species has been referred to as *Cotesia* jft09 in other papers (e.g. Fernández-Triana et al. 2011, 2016), and it corresponds in BOLD to BIN BOLD:AAA6099. The species seems to be related to a complex of species, from both Europe and North America but for the time being is left as an undescribed species, until more studies of the Holarctic fauna are carried out.

#### Dolichogenidea sicaria

Taxon classificationAnimaliaHymenopteraBraconidae

(Marshall, 1885)

Fig. 8

##### Distribution.

NEA, PAL.

**Figure 8.**
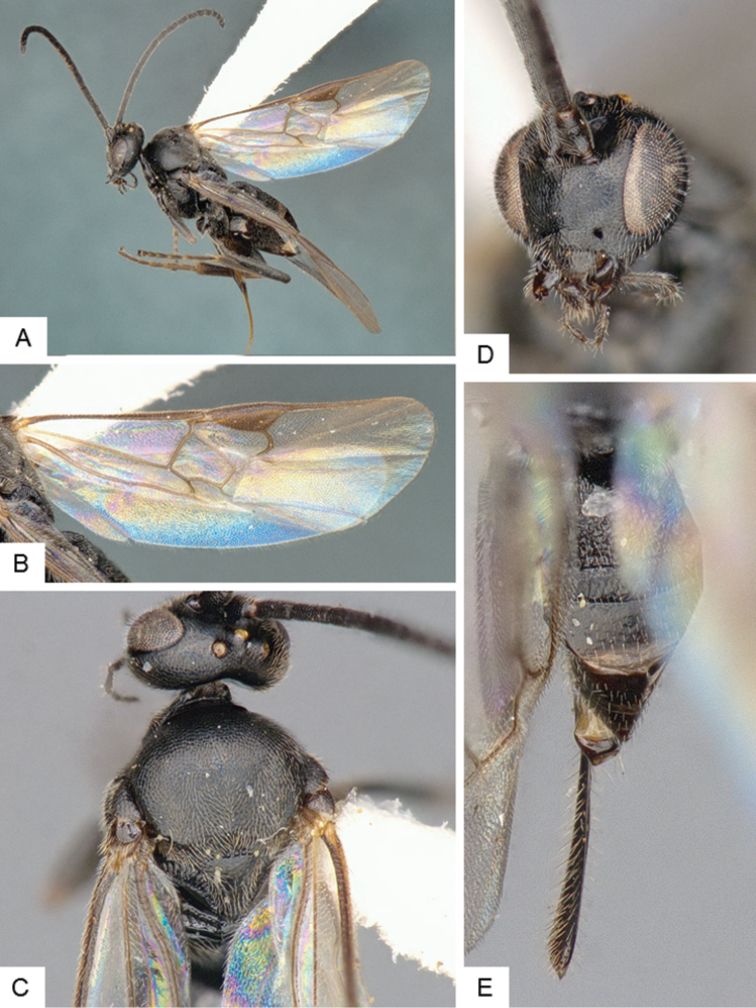
*Dolichogenidea sicaria*. **A** Habitus, lateral **B** Fore wing **C** Head and mesosoma (partially), dorsal **D** Head, frontal **E** Metasoma (partially) and ovipositor, dorsal.

##### Notes.

This species is widely distributed in the Holarctic region, and it has also been introduced into New Zealand (Yu et al. 2016). Here we record the species for the first time in the High Arctic: Greenland, as well as Axel Heiberg, Baffin and Ellesmere Islands. Várkonyi and Roslin (2013) and Wirta et al. (2016) recorded it as ‘*Dolichogenidea* sp.’ from Greenland. The sequence of that specimen in BOLD (sequence code: GRAFW237-11) matches several sequences of *Dolichogenidea sicaria* (from Canada, Norway, Sweden and USA specimens), clearly indicating that the Greenland specimen is conspecific with them. Hosts: In the High Arctic, Várkonyi and Roslin (2013) mentioned as probably host *Stenoptilia islandica* (Staudinger, 1857) (Pterophoridae), a record we accept here as very likely based on their explanation [Várkonyi and Roslin (2013) wrote: “On 17 July 2011, a microgastrine cocoon attached to the remains of a microlepidoptera larva was found under a tuft of *Saxifraga cespitosa* Linnaeus (Saxifragaceae) >700m in the bare basalt cap area of Aucellabjerg. By 24 August 2011, a female *Dolichogenidea* species hatched from this sample. As *S. cespitosa* is the host plant of *Stenoptilia islandica* (Staudinger) (Lepidoptera: Pterophoridae) (table 3), as several specimens of this microlepidopteran species were seen and collected (exclusively) at high elevations on Aucellabjerg, and as *Dolichogenidea* species (like all microgastrine wasps; for the Zackenberg species see Table 1) are koinobiont endoparasitoids of Lepidoptera larvae (Shaw and Huddleston 1991), *S. islandica* seems a potential host of this species. Clearly, direct rearing records are needed to verify this hypothesis.”]. In more southern localities, outside of the High Arctic, many other species of Lepidoptera have been cited as hosts of *D. sicaria* (e.g., Yu et al. 2016), with some of those records being questionable.

#### Dolichogenidea

Taxon classificationAnimaliaHymenopteraBraconidae

sp. 1

Fig. 9

##### Distribution.

NEA. Probably a High Arctic endemic.

**Figure 9.**
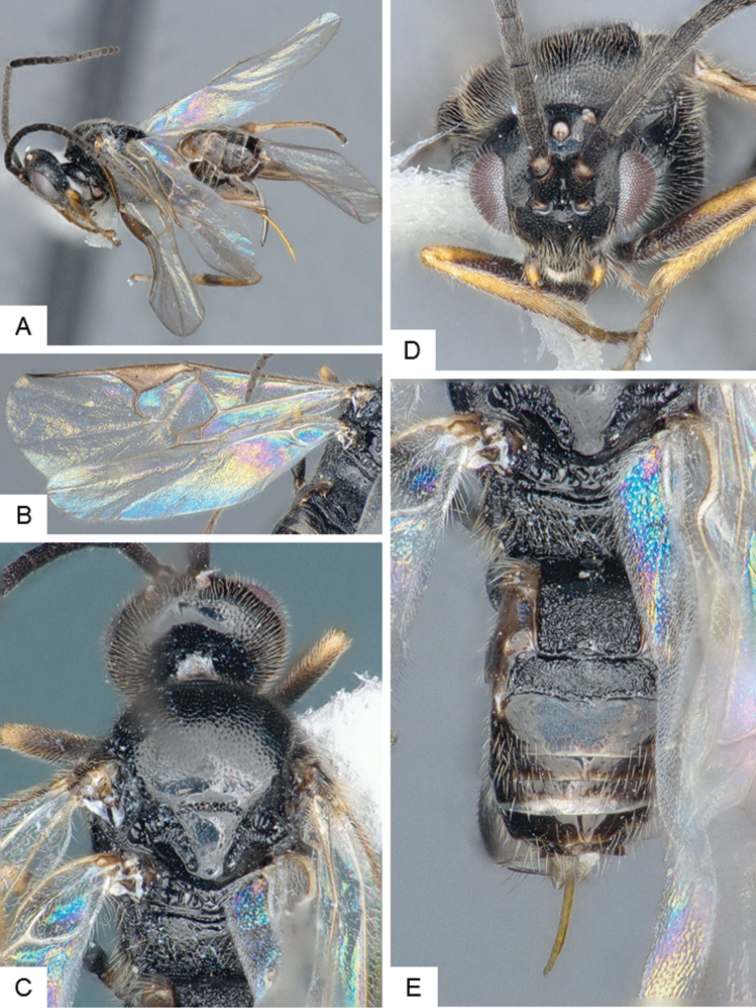
*Dolichogenidea* sp. 1. **A** Habitus, lateral **B** Fore wing and hind wing **C** Head and mesosoma, dorsal **D** Head, frontal-dorsal **E** Metasoma, dorsal.

##### Notes.

A total of 105 specimens from Banks and Baffin Islands. Differences in DNA barcodes, and morphology (sculpture of propodeum, mediotergites 1 and 2, length of fore wing vein R1), separate this species from the next one. The available DNA sequences for this species correspond in BOLD to BIN BOLD:AAE6509.

#### Dolichogenidea

Taxon classificationAnimaliaHymenopteraBraconidae

sp. 2

Fig. 10

##### Distribution.

NEA. Probably a High Arctic endemic.

**Figure 10.**
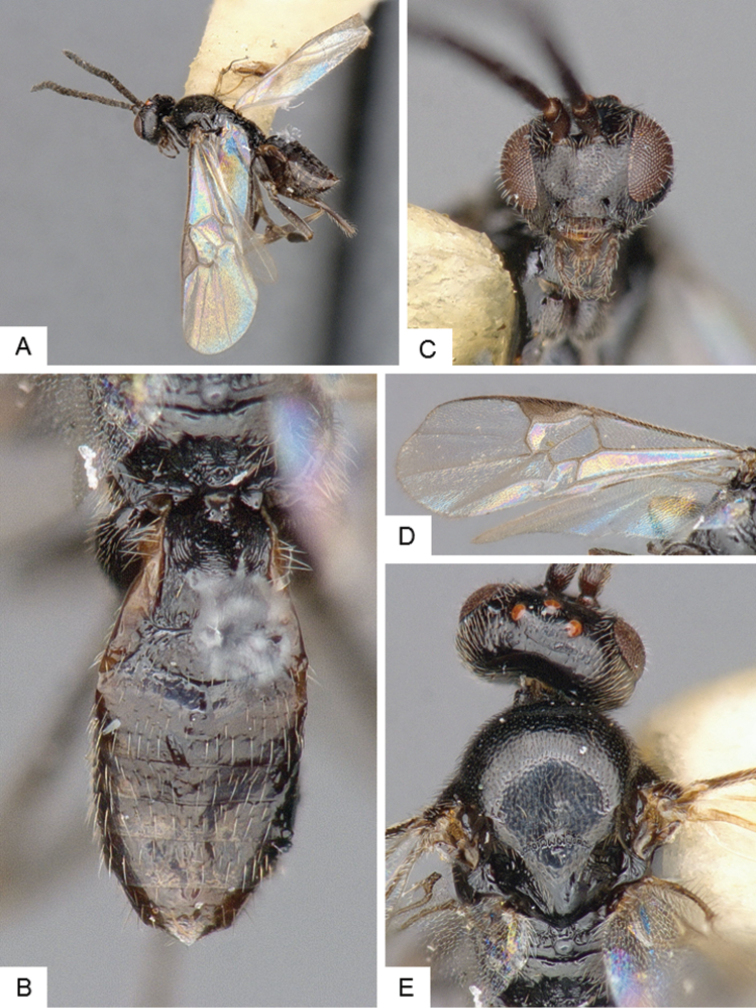
*Dolichogenidea* sp. 2. **A** Habitus, lateral **B** Metasoma, dorsal **C** Head, frontal **D** Fore wing **E** Head and mesosoma (partially), dorsal.

##### Notes.

A total of 21 specimens from Banks Island, see above for differences with previous species. Only a mini barcode (144 base pairs) is available from this species (from specimen with voucher code MIC 000290), which is not enough to clearly characterize the species from a DNA barcoding perspective.

#### Dolichogenidea

Taxon classificationAnimaliaHymenopteraBraconidae

sp. 3

Fig. 11

##### Distribution.

NEA. Probably a High Arctic endemic.

**Figure 11.**
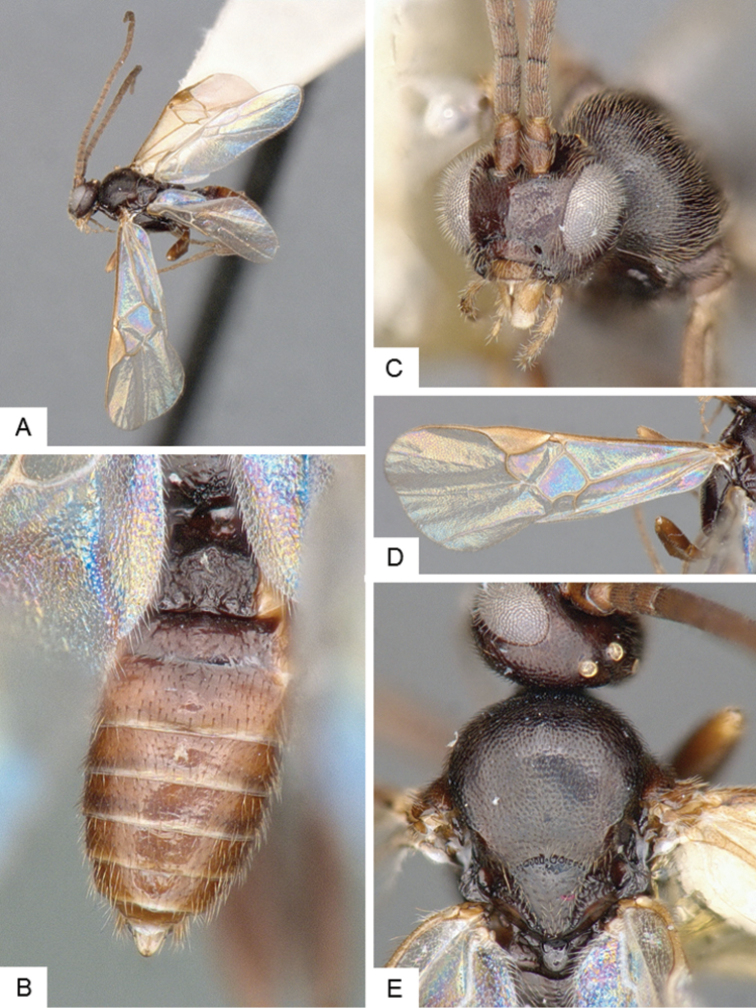
*Dolichogenidea* sp. 3. **A** Habitus lateral **B** Metasoma, dorsal **C** Head, frontal-lateral **D** Fore wing **E** Head (partially) and mesosoma (partially), dorsal.

##### Notes.

Three male specimens from Victoria Island. Although male specimens are usually less informative in terms of the taxonomy of Microgastrinae wasps, the studied specimens are very distinctive due to their very smooth propodeum and different shape and sculpture of mediotergites 1 and 2, as compared to the previous three species of *Dolichogenidea*. Thus, we consider them as a separate species. No DNA sequences are available for this species.

#### Glyptapanteles compressiventris

Taxon classificationAnimaliaHymenopteraBraconidae

(Muesebeck, 1921)

Fig. 12

##### Distribution.

NEA, PAL.

**Figure 12.**
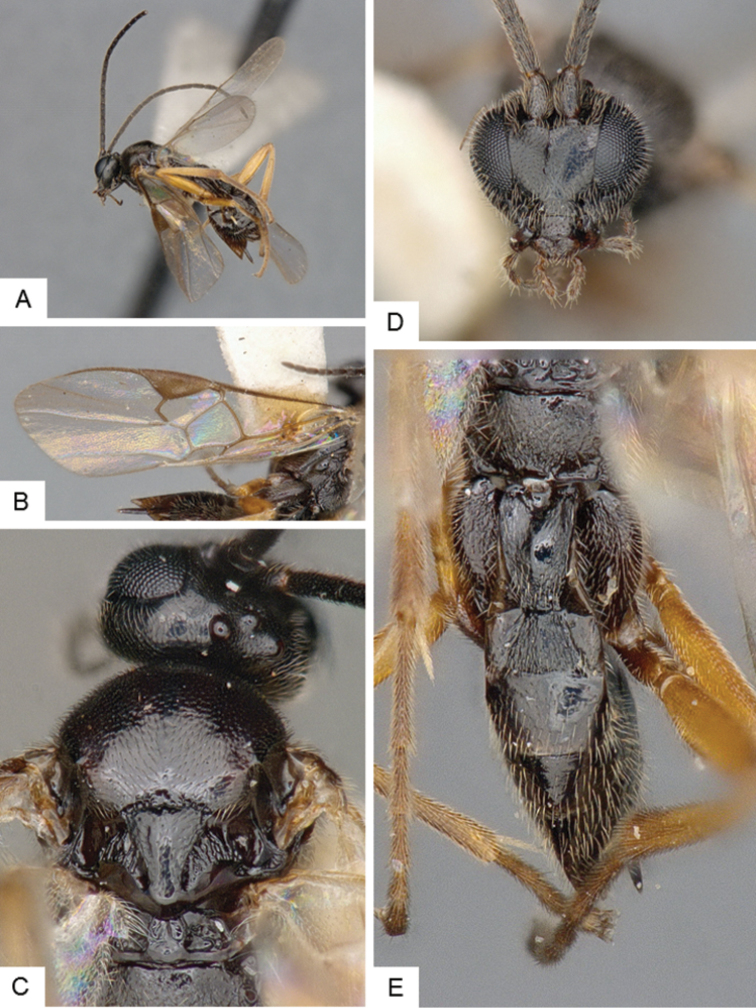
*Glyptapanteles compressiventris*. **A** Habitus, lateral **B** Fore wing **C** Head and mesosoma (partially), dorsal **D** Head, frontal **E** Metasoma, dorsal.

##### Notes.

A total of 14 specimens from Baffin and Dorset Islands. Only Clyde River (Clyde Inlet) can be considered northern (70° 29’ N); the other localities are from southern Baffin Island and Dorset Island (62–64° N). There are many specimens in the CNC from more southern Canadian localities, suggesting that this species is likely more common in southern Nearctic areas and that the CAA is the northernmost limit of the species’ range. Available barcodes suggest that the name *compressiventris* may include at least two cryptic species, but that is beyond the scope of this paper and thus for now all Canadian specimens are left under that name. The available DNA sequences for this species correspond in BOLD to BIN BOLD:ACE5800.

#### Glyptapanteles fulvipes

Taxon classificationAnimaliaHymenopteraBraconidae

(Haliday, 1834)

Fig. 13

##### Distribution.

NEA, PAL.

**Figure 13.**
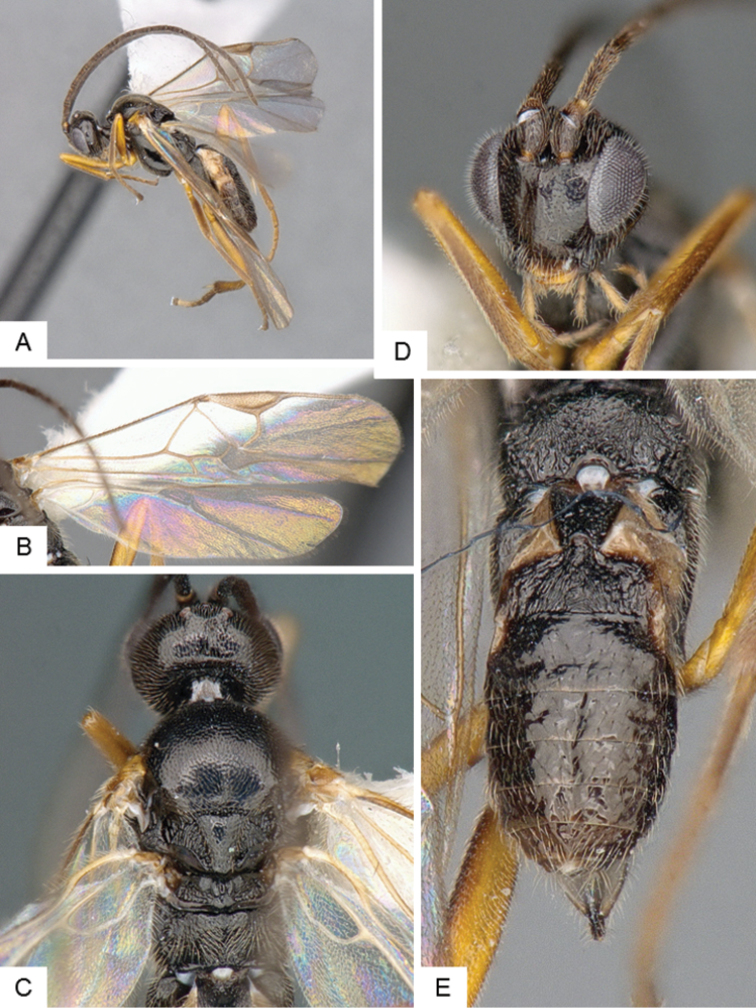
*Glyptapanteles fulvipes*. **A** Habitus, lateral **B** Fore wing and hind wing **C** Head and mesosoma, dorsal **D** Head, frontal **E** Metasoma, dorsal.

##### Notes.

A total of 179 specimens in total from Greenland and Axel Heiberg, Baffin, Ellesmere and Victoria Islands. The majority of the specimens identified in BOLD as *G. fulvipes* correspond to BIN BOLD:ACE7221 (but see next species for comments of a potential species complex).

#### Glyptapanteles pallipes

Taxon classificationAnimaliaHymenopteraBraconidae

(Reinhard, 1880)

##### Distribution.

NEA, OTL, PAL.

##### Notes.

This species is widely distributed in North America, Europe and Asia, usually from more southern areas, but also recorded from Greenland by Papp (1989). No other specimen has been found in the region since, neither by van Achterberg (2006) nor by us. Specimens deposited in the CNC (from southern localities) have been sampled for DNA barcoding and their sequences are similar to those of *G. fulvipes*. It seems likely that specimens previously identified and named as *G. pallipes* or *G. fulvipes* actually comprise a complex of morphologically cryptic species (e.g., see next species below). Solving that complex is beyond the scope of this paper.

#### Glyptapanteles

Taxon classificationAnimaliaHymenopteraBraconidae

sp. 1

Fig. 14

##### Distribution.

NEA. Probably a High Arctic endemic.

**Figure 14.**
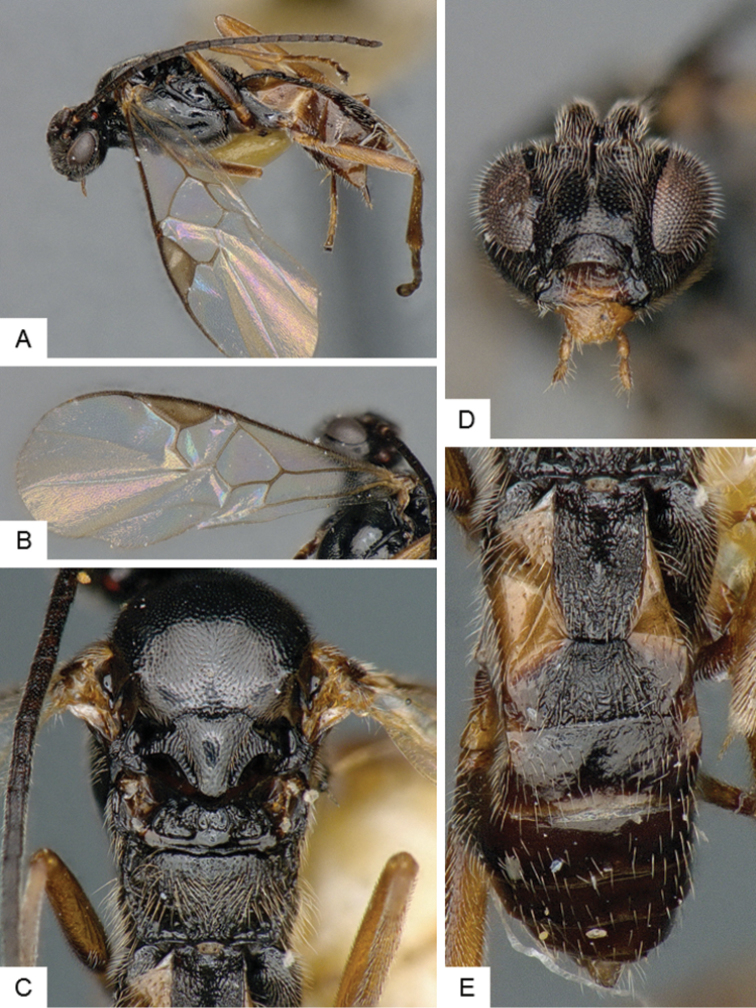
*Glyptapanteles* sp. 1. **A** Habitus, lateral **B** Fore wing **C** Mesosoma, dorsal **D** Head, frontal **E** Metasoma, dorsal.

##### Notes.

This species is morphologically related to *G. fulvipes* and *G. pallipes*. Slight differences in morphology and partial DNA barcodes (but only mini barcodes of 144 base pairs are available from High Arctic specimens) suggest this is a different species. However, it cannot be described until a comprehensive study of the *fulvipes*/*pallipes* complex is done. Most of the 46 studied specimens are from Banks Island, with two specimens from Bylot and Baffin Islands.

#### Glyptapanteles

Taxon classificationAnimaliaHymenopteraBraconidae

sp. 2

Fig. 15

##### Distribution.

NEA. High Arctic and some additional, unpublished records in BOLD from northern Canada (mainland).

**Figure 15.**
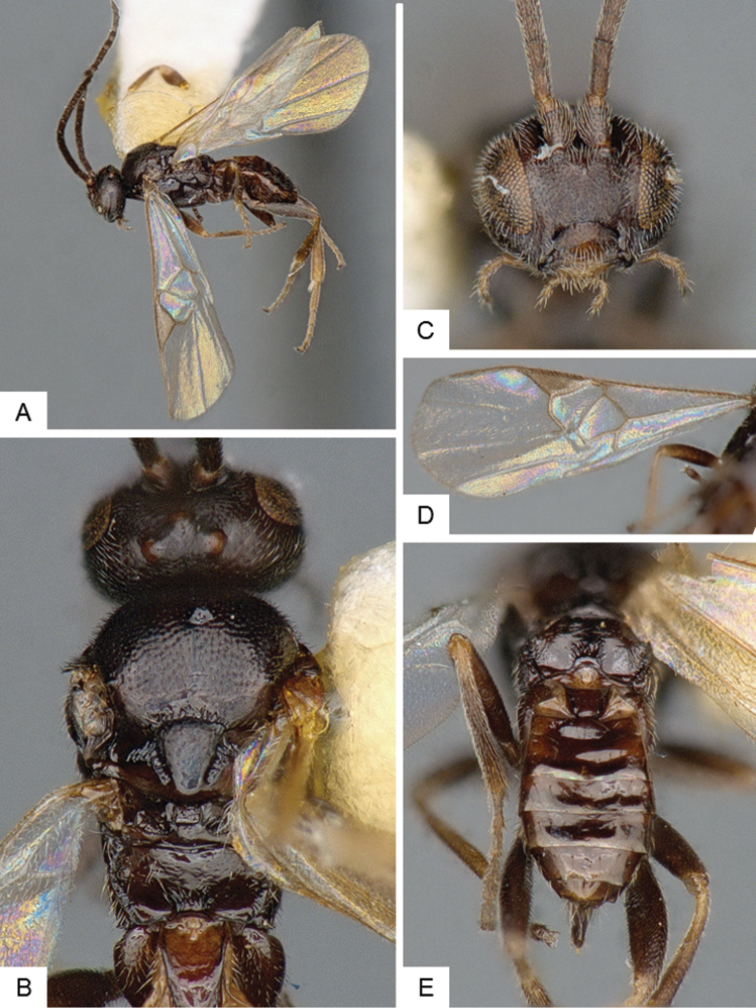
*Glyptapanteles* sp. 2. **A** Habitus, lateral **B** Head and mesosoma, dorsal **C** Head, fontal **D** Fore wing **E** Metasoma, dorsal.

##### Notes.

Most specimens from Baffin Island (Clyde Inlet), but one specimen from Peary Land (Greenland). They are characterized by almost completely smooth mediotergites 1 and 2. No DNA sequences are available. We have seen other specimens from localities in mainland Canada. Additional study of the whole Holarctic fauna of *Glyptapanteles* will be needed before the identity of this species can be established.

#### Glyptapanteles

Taxon classificationAnimaliaHymenopteraBraconidae

sp. 3

Fig. 16

##### Distribution.

NEA. Probably a High Arctic endemic.

**Figure 16.**
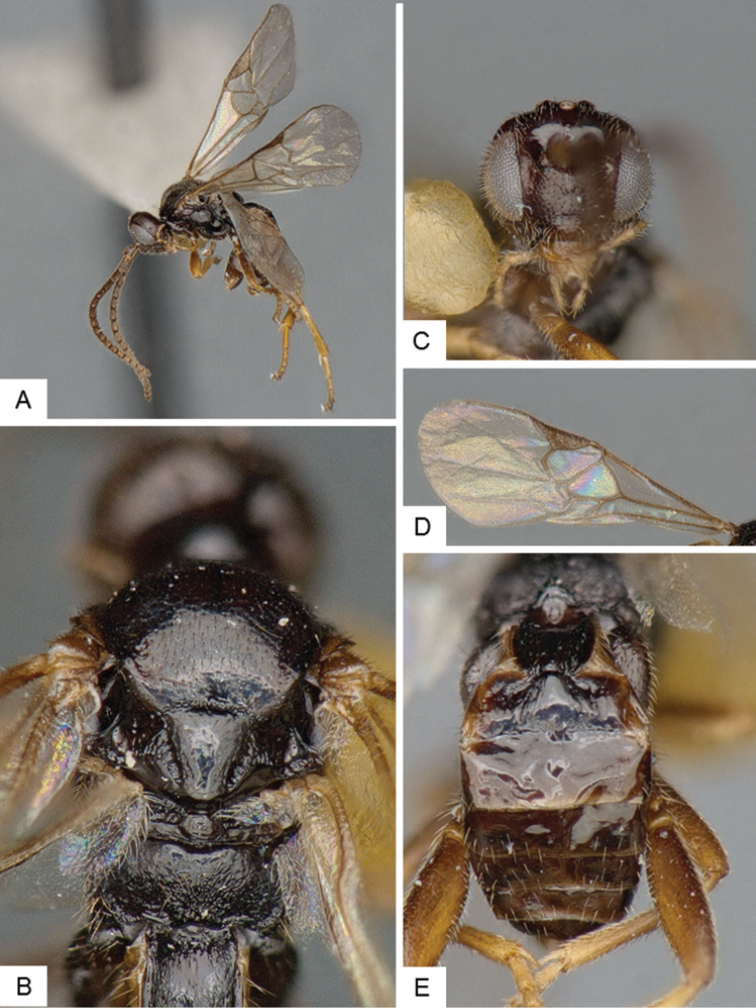
*Glyptapanteles* sp. 3. **A** Habitus, lateral **B** Mesosoma, dorsal **C** Head, frontal **D** Fore wing **E** Metasoma, dorsal.

##### Notes.

One female from Banks Island. Much more sculptured mediotergites 1 and 2 than in any other High Arctic species of *Glyptapanteles*. We are also including here three specimens from Banks Island (Aulavik National Park) that we were not be able to examine, but the available picture in BOLD is similar enough to the female specimen to place them here, at least provisionally. The available DNA sequences for this species correspond in BOLD to BIN BOLD:ACR4201.

#### Glyptapanteles

Taxon classificationAnimaliaHymenopteraBraconidae

sp. 4

Figs 17 , 18

##### Distribution.

NEA. Probably a High Arctic endemic.

**Figure 17.**
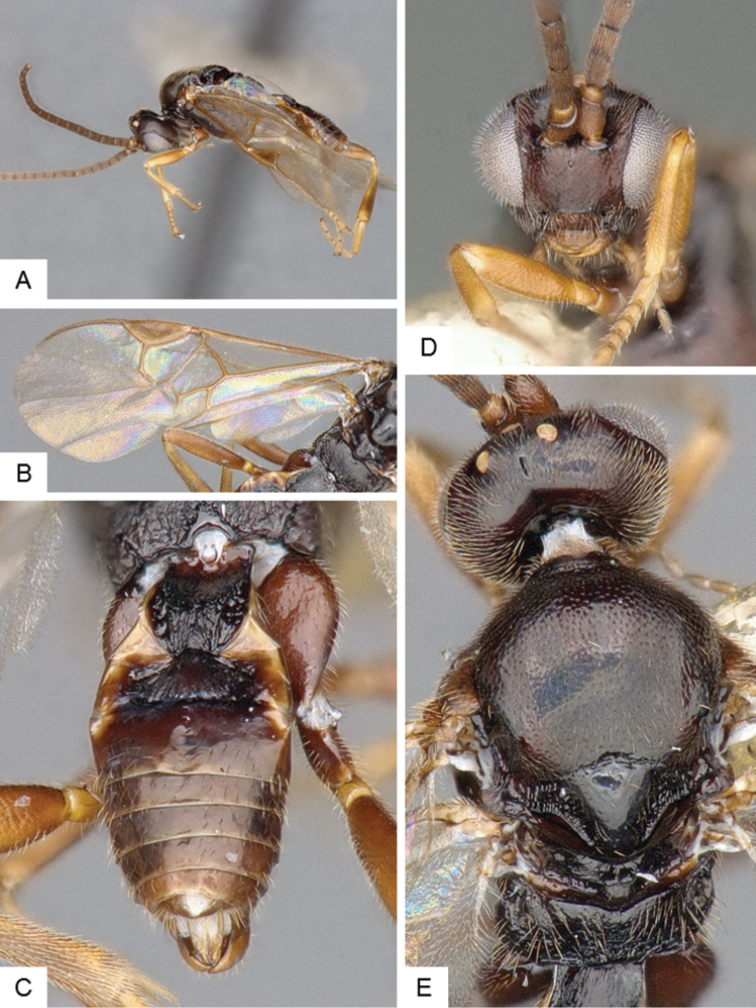
*Glyptapanteles* sp. 4. **A** Habitus, lateral **B** Fore wing **C** Metasoma, dorsal **D** Head, frontal **E** Head and mesosoma, dorsal.

**Figure 18.**
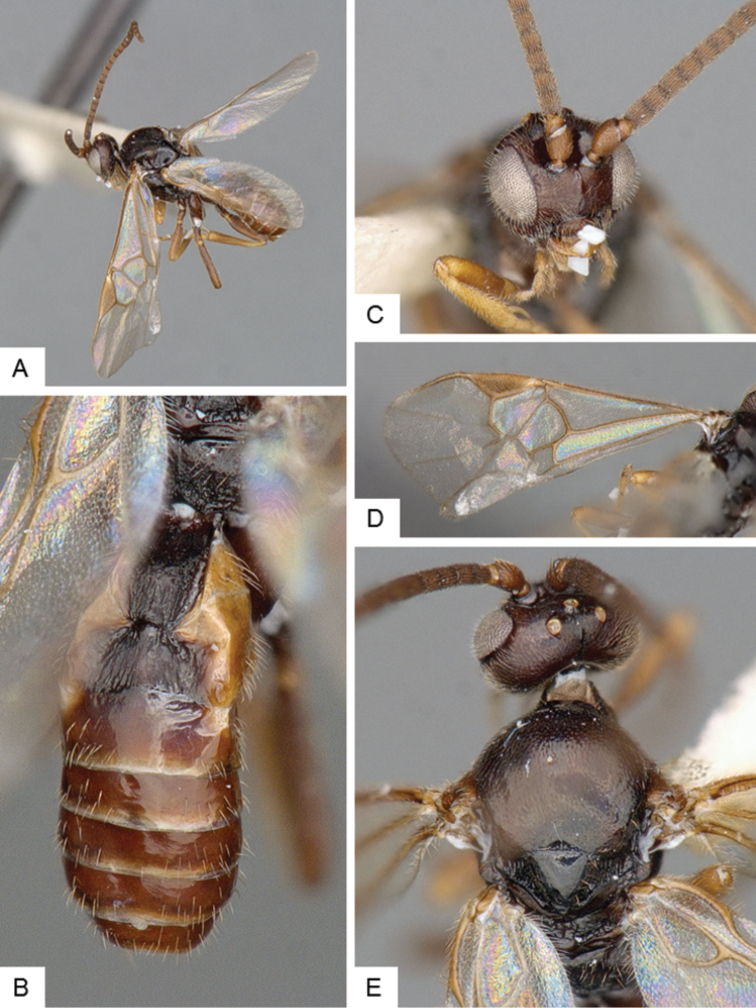
*Glyptapanteles* sp. 4. **A** Habitus, lateral **B** Metasoma, dorsal **C** Head, frontal **D** Fore wing **E** Head and mesosoma (partially), dorsal.

##### Notes.

Four female and 23 male specimens, mostly collected in Victoria Island, with some from Baffin Island (Clyde River). The external genitalia of male specimens suggest that this species might better be placed within *Sathon* (which would represent the northernmost record for that genus); however, the ovipositor and ovipositor sheaths in females indicate it is better placed within *Glyptapanteles*. DNA barcodes could only be obtained from three male specimens, but the sequences were too short (104-144 base pairs) and thus DNA barcoding could not conclusively place the species within any of the two potential genera. Based on the length of the female ovipositor we are provisionally placing this species within *Glyptapanteles*, although this may change with future studies.

#### Glyptapanteles

Taxon classificationAnimaliaHymenopteraBraconidae

sp. 5

Fig. 19

##### Distribution.

NEA. High Arctic endemic.

**Figure 19.**
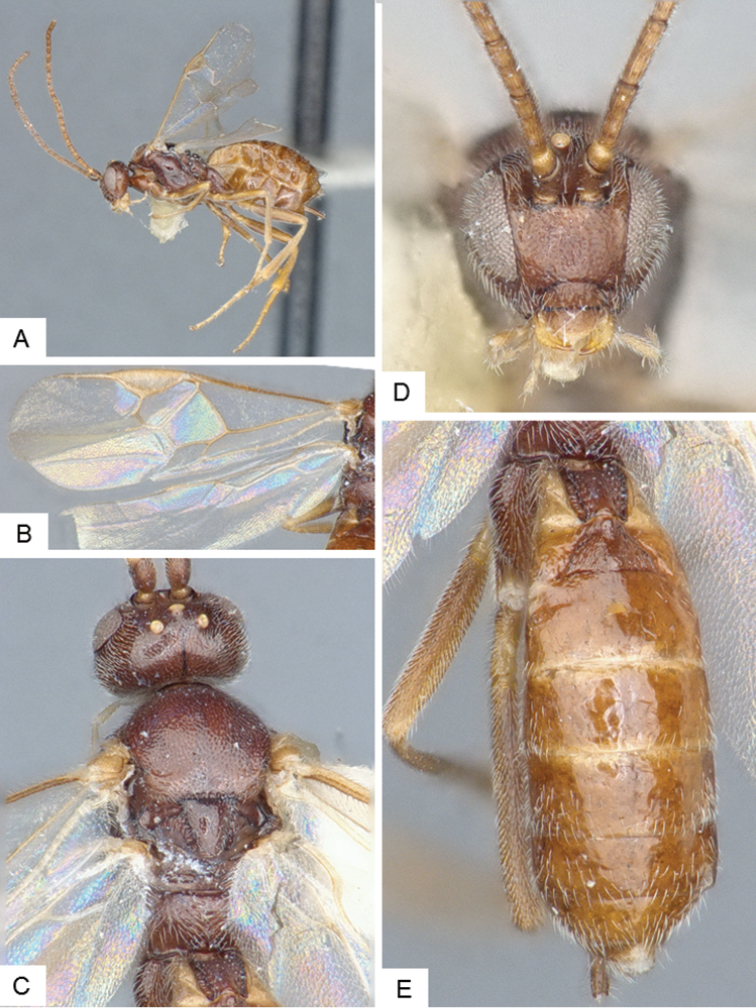
*Glyptapanteles* sp. 5. **A** Habitus, lateral **B** Fore wing and hind wing (partially) **C** Head and mesosoma, dorsal **D** Head, frontal **E** Metasoma, dorsal.

##### Notes.

Collected in Alert (during three different time periods: 1951, 2001 and 2008) and Hazen Camp (1963), both on Ellesmere Island. There are also two specimens from Greenland, one collected in 1966, and the other between 2009–2011 (no clear date established, see Várkonyi and Roslin 2013). Additionally, in the CNC collection there is a pin with host remnants and the wasp cocoons, clearly indicating that the parasitoid is a gregarious species. There are five full DNA barcodes from the 2008 samples, as well as three mini barcodes (134–144 base pairs) from specimens collected in 1951 which perfectly match the full barcode sequences. *Glyptapanteles* sp. 5 has 11 base pairs of difference (1.7 %) with the rest of the *fulvipes* (or near fulvipes) barcoded specimens that are available in BOLD from all over the Holarctic, and the new species cluster is clearly distinct. Host: *Polia richardsoni* (Curtis, 1834) (Noctuidae), this is the second record of a Microgastrinae parasitoid for that Lepidoptera species (recently *Microplitis lugubris* had been reported from Greenland by Wirta et al. (2014)). Because of the unique barcode and lepidopteran host, we consider this to be a new *Glyptapanteles* species, to be described in a separate paper. The available DNA sequences for this species correspond in BOLD to BIN BOLD:ABY9539.

#### Glyptapanteles

Taxon classificationAnimaliaHymenopteraBraconidae

sp. 6

Fig. 20

##### Distribution.

NEA. Probably a High Arctic endemic.

**Figure 20.**
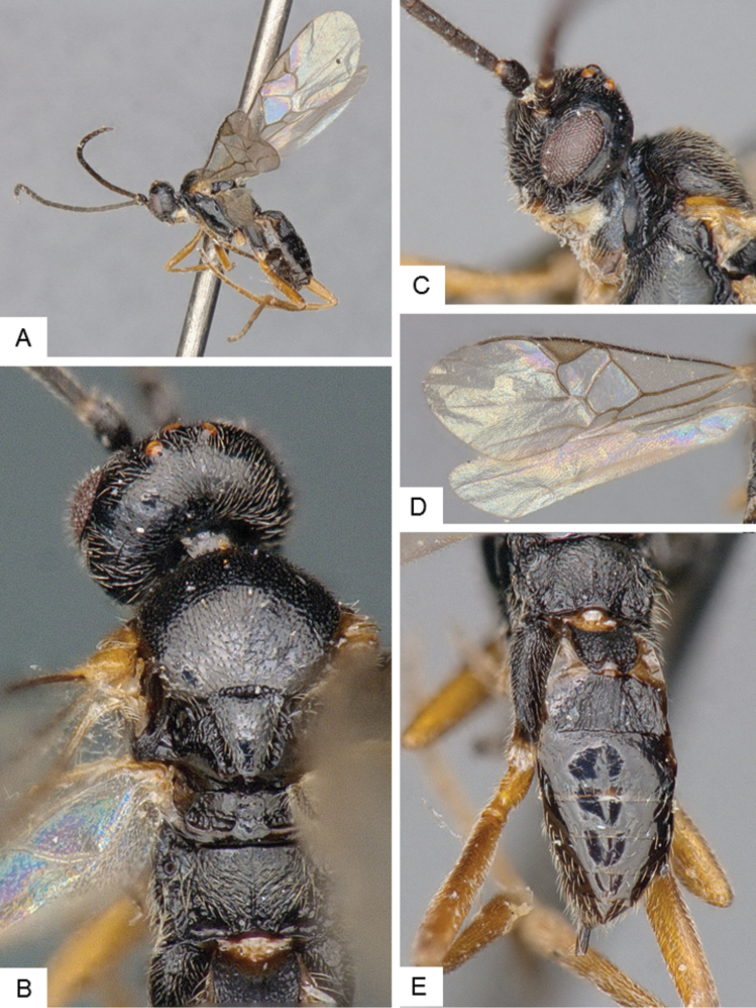
*Glyptapanteles* sp. 6. **A** Habitus, lateral **B** Head and mesosoma, dorsal **C** Head, lateral **D** Fore wing and hind wing **E** Metasoma, dorsal.

##### Notes.

Two female and two male specimens from Ellesmere Island. The wing venation is strikingly different from all other *Glyptapanteles* occurring in the High Arctic.

#### Illidops

Taxon classificationAnimaliaHymenopteraBraconidae

sp. 1

Fig. 21

##### Distribution.

NEA. Probably a High Arctic endemic.

**Figure 21.**
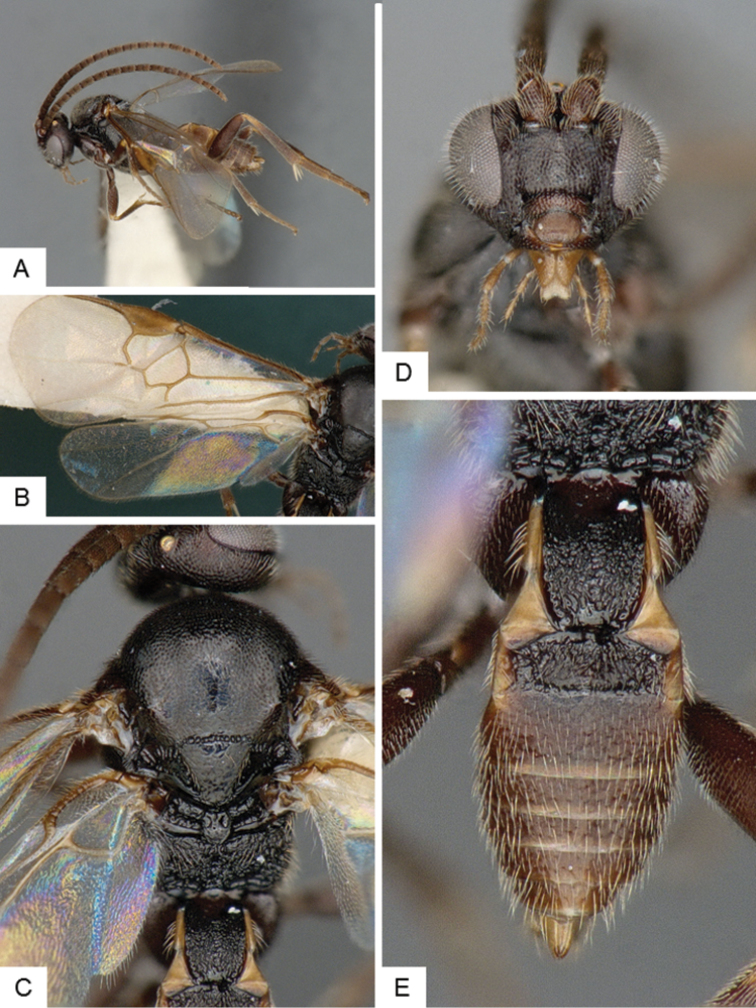
*Illidops* sp. 1. **A** Habitus, lateral **B** Fore wing and hind wing **C** Head and mesosoma, dorsal **D** Head, frontal **E** Metasoma, dorsal.

##### Notes.

Greenland, Peary Land. One female specimen (voucher code MIC000287), with a mini barcode of 144 base pairs. DNA barcoding and slight morphological differences separate this species from the following one.

#### Illidops

Taxon classificationAnimaliaHymenopteraBraconidae

sp. 2

Fig. 22

##### Distribution.

NEA. Probably a High Arctic endemic.

**Figure 22.**
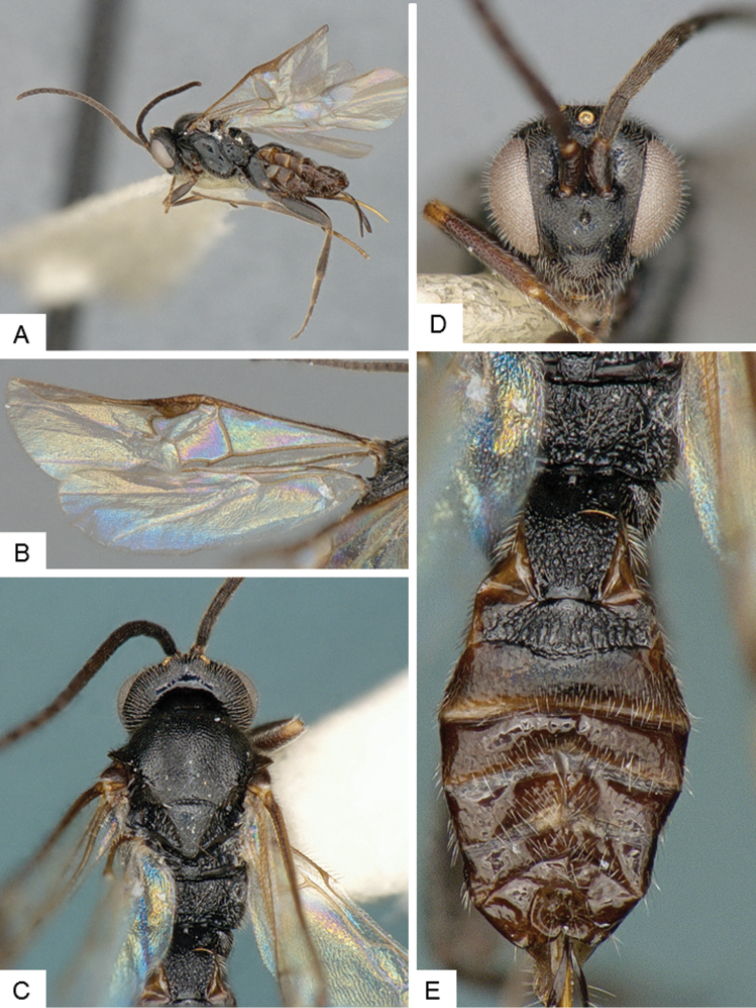
*Illidops* sp. 2. **A** Habitus, lateral **B** Fore wing and hind wing **C** Mesosoma, dorsal **D** Head, frontal **E** Metasoma, dorsal.

##### Notes.

One female and 16 male specimens from Baffin, Devon, Melville and Victoria Islands. The two available mini barcodes (126–144 base pairs) separate this species from the Greenlandic species of *Illidops*.

#### Microgaster

Taxon classificationAnimaliaHymenopteraBraconidae

sp. 1

Fig. 23

##### Distribution.

NEA.

**Figure 23.**
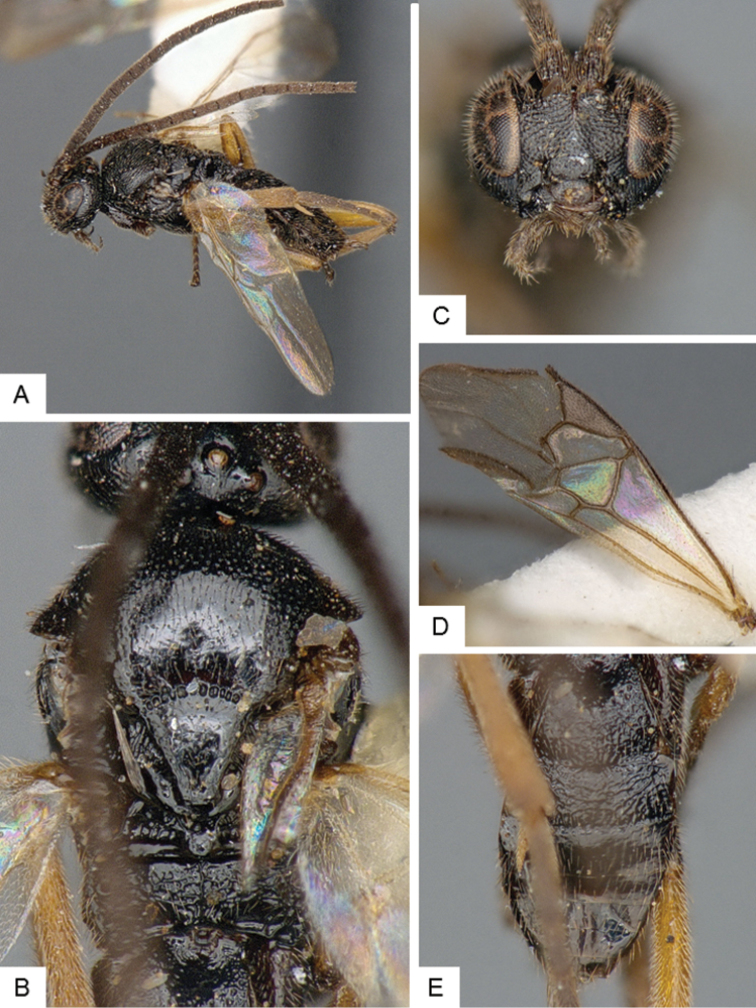
*Microgaster* sp. 1. **A** Habitus, lateral **B** Mesosoma, dorsal **C** Head, frontal **D** Fore wing **E** Metasoma (partially), dorsal.

##### Notes.

One male specimen from Banks Island (voucher code MIC000311). The poor condition of the specimen prevents further identification. Its associate sequence (a mini barcode of 144 base pairs) is not sufficient for an unambiguous placement of the species within other *Microgaster* sequences in BOLD.

#### Microplitis coactus

Taxon classificationAnimaliaHymenopteraBraconidae

(Lundbeck, 1896)

**Figure 24.**
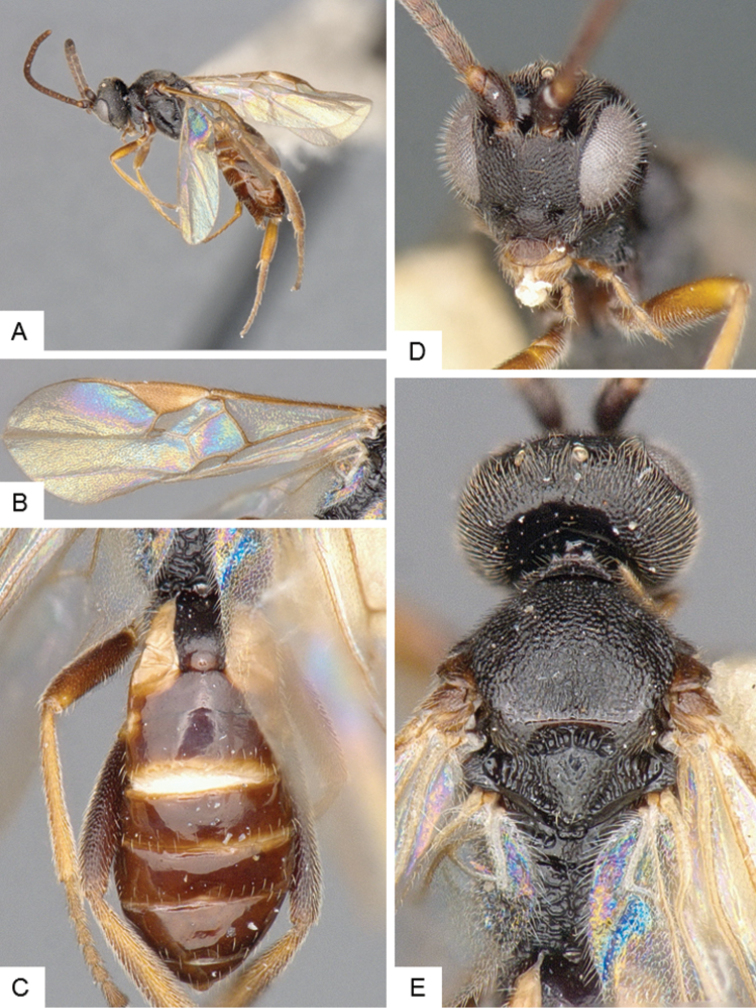
*Microplitis coactus*. **A** Habitus, lateral **B** Fore wing **C** Metasoma, dorsal **D** Head, dorsal **E** Head and mesosoma, dorsal.

**Figure 25.**
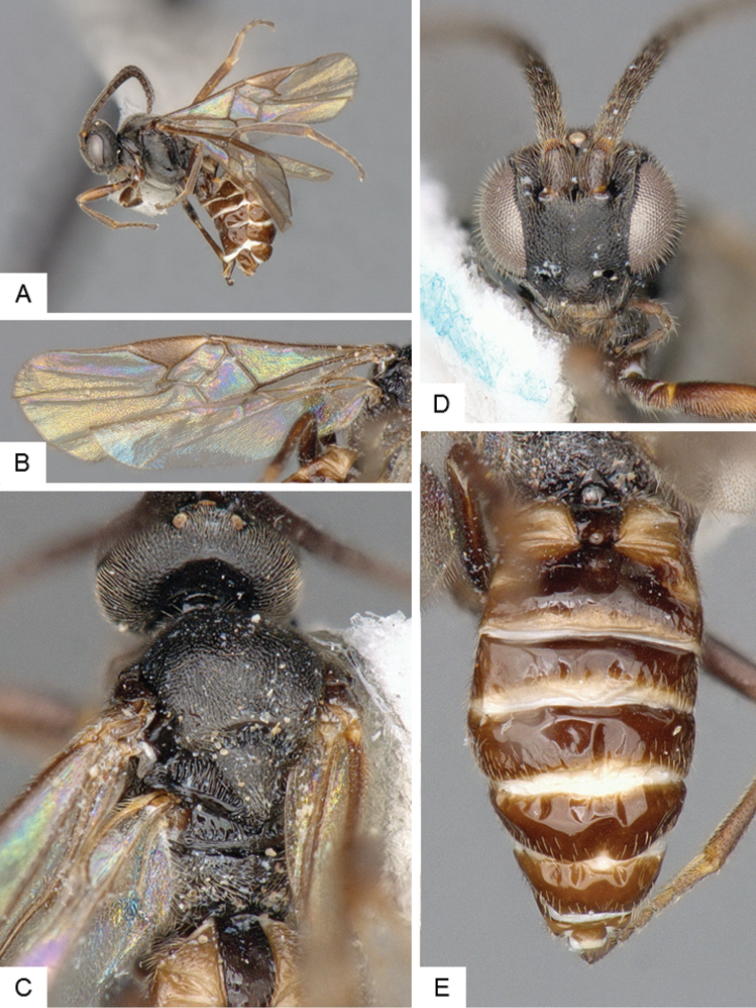
*Microplitis coactus*. **A** Habitus, lateral **B** Fore wing **C** Head and mesosoma, dorsal **D** Head, frontal **E** Metasoma, dorsal.

**Figure 26.**
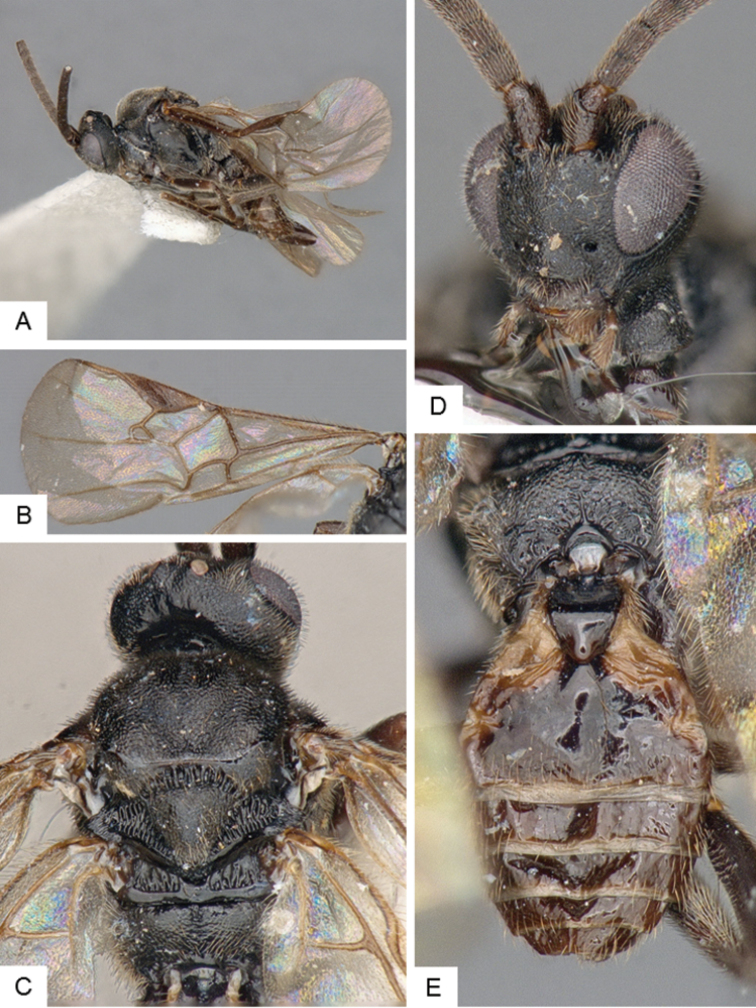
*Microplitis coactus*. **A** Habitus, lateral **B** Fore wing **C** Head and mesosoma, dorsal **D** Head, frontal **E** Metasoma and propodeum, dorsal.

**Figure 27.**
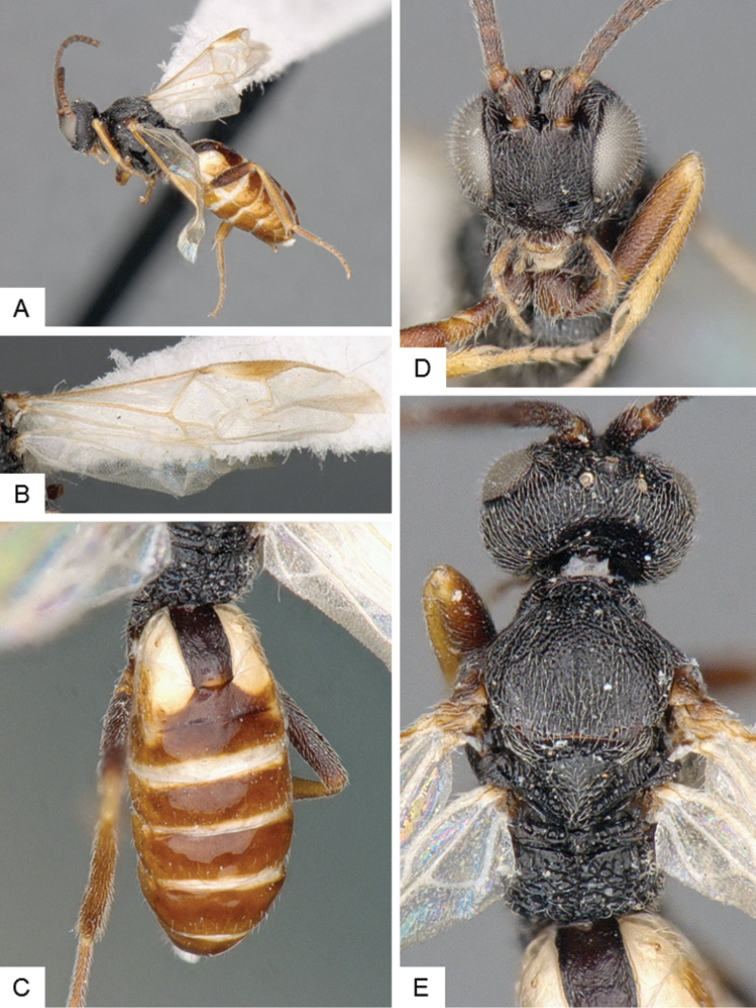
*Microplitis coactus*. **A** Habitus, lateral **B** Fore wing **C** Metasoma, dorsal **D** Head, frontal **E** Head and mesosoma, dorsal.

Figs 24 , 25 , 26 , 27

##### Distribution.

NEA, PAL.

##### Notes.

A total of 35 specimens from Devon and Ellesmere Islands, as well as Greenland. The Canadian specimens match the available descriptions provided by Papp (1984) and van Achterberg (2006), but the metafemur is not as thick as mentioned for the Greenlandic and Icelandic specimens. However, all the other morphological characters mentioned by those authors agree with the specimens from the CAA, so for the time being, we are considering them all to be conspecific. One female specimen from Devon Island and one male specimen from Ellesmere Island (voucher codes MIC 000313 & MIC 000315) have mini barcodes (114–144 base pairs), although they differ rather substantially (by seven base pairs) and it is not sufficient to unambiguously place these specimens within other sequences of *Microplitis* in BOLD. Hosts: *Noctua* sp. (Noctuidae).

#### Microplitis lugubris

Taxon classificationAnimaliaHymenopteraBraconidae

(Ruthe, 1860)

Fig. 28

##### Distribution.

NEA, PAL.

**Figure 28.**
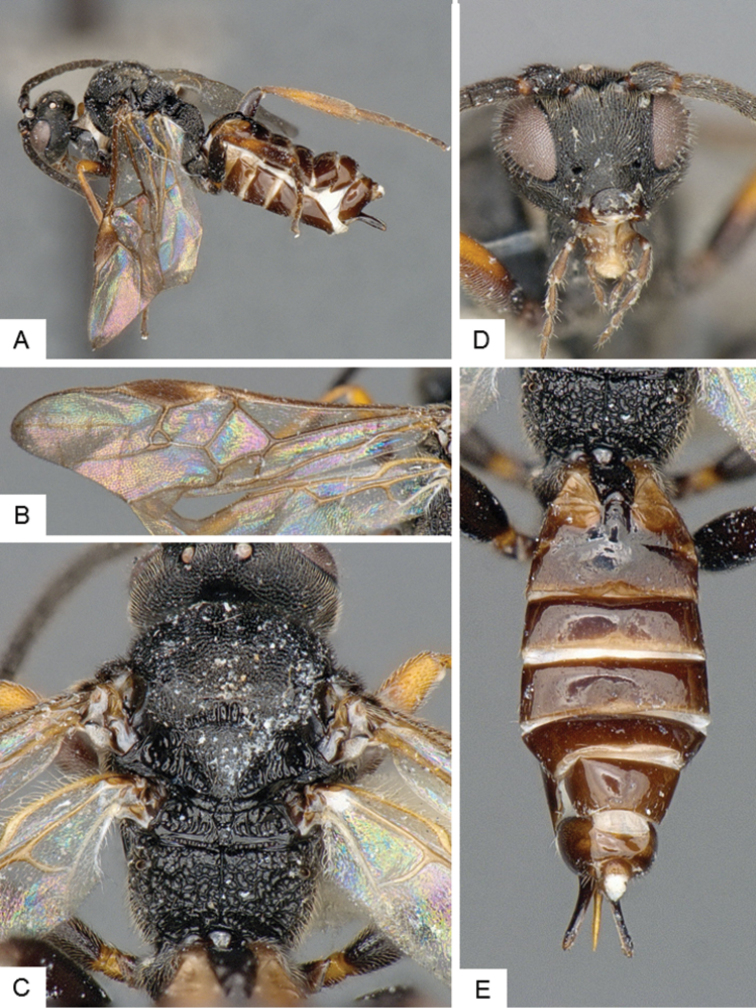
*Microplitis lugubris*. **A** Habitus, lateral **B** Fore wing **C** Mesosoma, dorsal **D** Head, frontal **E** Metasoma, dorsal.

##### Notes.

The only Nearctic record until now was from Greenland (van Achterberg 2006, Várkonyi and Roslin 2013). Here it is recorded for the first time from Canada (Ellesmere Island) as well as an additional locality record for Greenland (Peary Land, based on one specimen deposited in the CNC). We are also aware of specimens from a southern Canadian locality: Churchill, Manitoba (at around 59° N), which had been named as “*Microplitis* jft01” in previous papers (Fernández-Triana 2010, Fernández-Triana et al. 2011). The records from Churchill expand considerably the southernmost distribution of the species within the Nearctic. Based on the number of specimens (716), *Microplitis lugubris* is probably the most commonly found species of Microgastrinae in Greenland (although most of the specimens came from rearing caterpillars, see Várkonyi and Roslin 2013). The available DNA sequences for this species correspond in BOLD to BIN BOLD:ABY9068.

#### Microplitis sp. near lugubris

Taxon classificationAnimaliaHymenopteraBraconidae

Fig. 29

##### Distribution.

NEA. High Arctic and some additional, unpublished records in BOLD from northern Canada (mainland).

**Figure 29.**
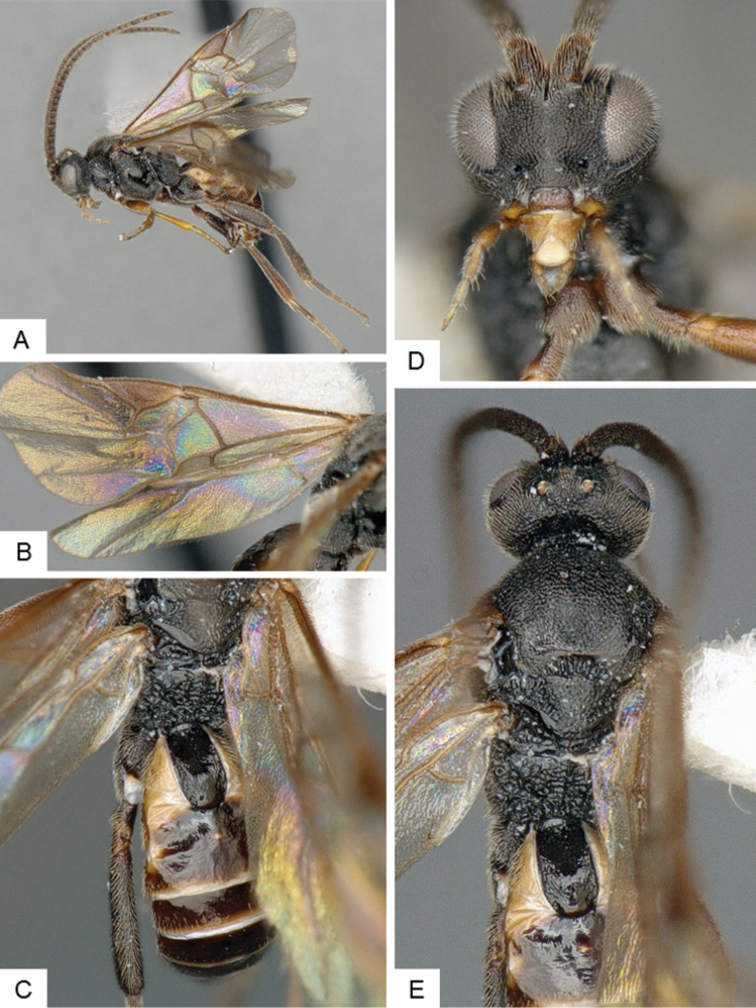
Microplitis sp. near lugubris. **A** Habitus, lateral **B** Fore wing and hind wing **C** Metasoma (partially), dorsal **D** Head, frontal **E** Head and mesosoma, dorsal.

##### Notes.

Five males from Bylot Island; we have also seen numerous specimens from Churchill, Manitoba, Canada (which have in BOLD the interim name “*Microplitis* jft04”). This species is morphologically similar to *M. lugubris*, but we consider it a different species based on the significant difference in the DNA barcodes (59 base pairs, representing 8.9% of differences in the DNA barcoding region). The available DNA sequences for this species correspond in BOLD to BIN BOLD:AAB1314.

#### Microplitis lugubroides

Taxon classificationAnimaliaHymenopteraBraconidae

van Achterberg, 2006

##### Distribution.

NEA. High Arctic endemic.

##### Notes.

Only known from the original description, from Greenland.

#### Microplitis mandibularis

Taxon classificationAnimaliaHymenopteraBraconidae

(Thomson, 1895)

##### Distribution.

NEA, PAL.

##### Notes.

The only record for the High Arctic is from Greenland (van Achterberg 2006).

#### Microplitis sofron

Taxon classificationAnimaliaHymenopteraBraconidae

Nixon, 1970

##### Distribution.

NEA, PAL.

##### Notes.

Recorded from Greenland, but considered a dubious record by van Achterberg (2006).

#### Micro plitis sp. nr. sofron

Taxon classificationAnimaliaHymenopteraBraconidae

Fig. 30

##### Distribution.

NEA. Probably a High Arctic endemic.

**Figure 30.**
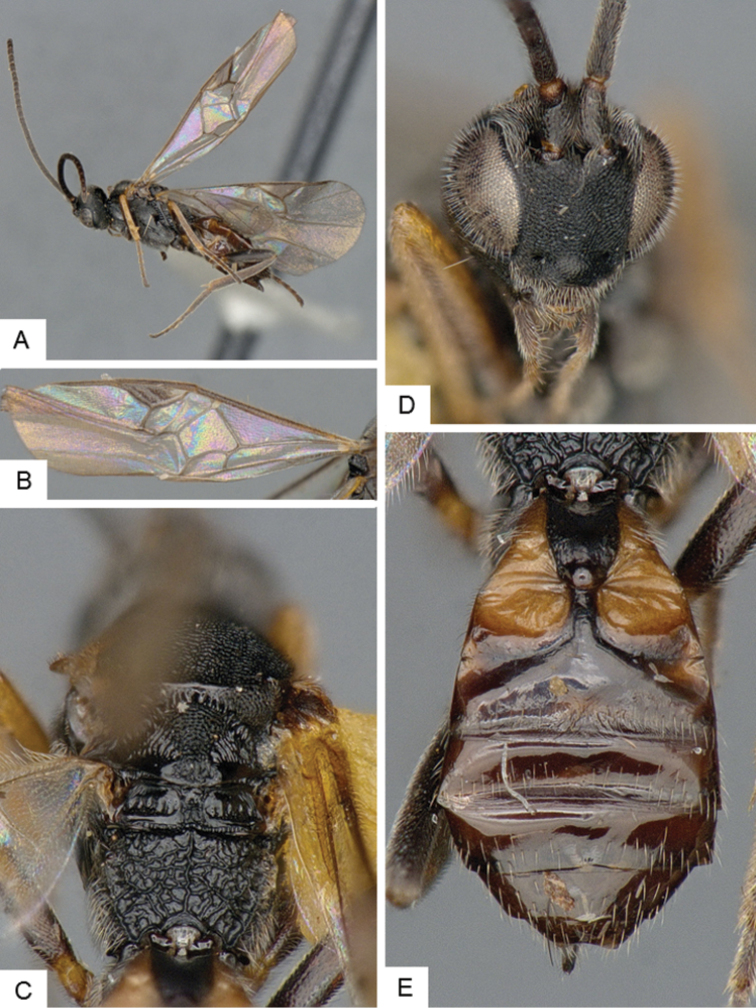
Microplitis sp. near sofron. **A** Habitus, lateral **B** Fore wing **C** Mesosoma, dorsal **D** Head, frontal **E** Metasoma, dorsal.

##### Notes.

A total of 12 specimens from Banks and Victoria Islands. This species will run to *M. sofron* in the keys provided by Nixon (1970), Papp (1984), and van Achterberg (2006). However, its metatibia is not bright yellow, and the shape of mediotergite 1 does not resemble the illustration of Papp (1984: figure 83). The color of metatibia and shape of mediotergite 1 are actually closer to *M. lugubroides*, but from that species it differs in the length of the last flagellomere, the main feature that van Achterberg used to separate *M. sofron* from *M. lugubroides*. The Canadian specimens probably represent a new species, but without examining the types of *sofron* and *lugubroides* we cannot be certain. No DNA sequences are available for this species.

### Aditional species

A few specimens, currently identified to genus level only, are likely to represent additional species records for the High Arctic. They are listed below, pending further study to assess their status.

*Cotesia* specimens from Greenland. Specimens with voucher codes ZMUC00023383, ZMUC00023385, ZMUC00023386, BIOUG15488-A02, 24361-A10, 24361-A12, 24361-B09, 24361-E07, 24388-C11, 24391-G12, 24412-H08, 24478-E01, 24523-C12, ZA2009-100, ZA2010-103, ZA2010-104, ZMUC00023387, ZMUC00023382, ZMUC00023381.

*Glyptapanteles* specimens from Baffin, Banks and Bylot Islands. Specimens with voucher codes BIOUG16577-D03, BIOUG16811-D10, CNCH0578, CNCH0579, CNCH0580, MIC000306, MIC000333.

## Supplementary Material

XML Treatment for Cotesia crassifemorata

XML Treatment for Cotesia eliniae

XML Treatment for Cotesia fascifemorata

XML Treatment for Cotesia hallii

XML Treatment for Cotesia yakutatensis

XML Treatment for Cotesia

XML Treatment for Cotesia

XML Treatment for Cotesia

XML Treatment for Cotesia

XML Treatment for Cotesia

XML Treatment for Dolichogenidea sicaria

XML Treatment for Dolichogenidea

XML Treatment for Dolichogenidea

XML Treatment for Dolichogenidea

XML Treatment for Glyptapanteles compressiventris

XML Treatment for Glyptapanteles fulvipes

XML Treatment for Glyptapanteles pallipes

XML Treatment for Glyptapanteles

XML Treatment for Glyptapanteles

XML Treatment for Glyptapanteles

XML Treatment for Glyptapanteles

XML Treatment for Glyptapanteles

XML Treatment for Glyptapanteles

XML Treatment for Illidops

XML Treatment for Illidops

XML Treatment for Microgaster

XML Treatment for Microplitis coactus

XML Treatment for Microplitis lugubris

XML Treatment for Microplitis sp. near lugubris

XML Treatment for Microplitis lugubroides

XML Treatment for Microplitis mandibularis

XML Treatment for Microplitis sofron

XML Treatment for Micro plitis sp. nr. sofron

## Supplementary materials

Supplementary material 1 Details of all studied specimens of Microgastrinae (Hymenoptera, Braconidae) from the Canadian Arctic Archipelago and Greenland Data type: speciems data This dataset is made available under the Open Database License (http://opendatacommons.org/licenses/odbl/1.0/). The Open Database License (ODbL) is a license agreement intended to allow users to freely share, modify, and use this Dataset while maintaining this same freedom for others, provided that the original source and author(s) are credited.Jose Fernández-Triana, Joel Buffam, Melanie Beaudin, Hannah Davis, Ana Fernández-Galliano, Emily Griffin, Shang-Yao Lin, Megan K. McAulay, Megan K. McAulay, Robin Richter, Freddy Rodriguez, Gergely Várkonyi

Supplementary material 2 R code used to generate the map Data type: codes This dataset is made available under the Open Database License (http://opendatacommons.org/licenses/odbl/1.0/). The Open Database License (ODbL) is a license agreement intended to allow users to freely share, modify, and use this Dataset while maintaining this same freedom for others, provided that the original source and author(s) are credited.Jose Fernández-Triana, Joel Buffam, Melanie Beaudin, Hannah Davis, Ana Fernández-Galliano, Emily Griffin, Shang-Yao Lin, Megan K. McAulay, Megan K. McAulay, Robin Richter, Freddy Rodriguez, Gergely Várkonyi
