# Supplementary material for: An annotated and illustrated checklist of Microgastrinae wasps (Hymenoptera, Braconidae) from the Canadian Arctic Archipelago and Greenland
Source: Zookeys. 2017 Aug 17;(691):49–101. doi: 10.3897/zookeys.691.14491 (PMC5672697; doi:10.3897/zookeys.691.14491)
Supplement: Supplementary material 2 — R code used to generate the map [file zookeys-691-049-s002.docx]

**R code used to generate the map**

# Required packages

library(rgdal)

library(raster)

# Specify working directory (on Acer laptop)

setwd("C:/Users/Peter/Documents")

# Import CSV file with coordinates

map.coor <- read.csv("List.of.localities.with.Lat.Long.csv", header=T)

# Import tif file

# Note: raster file was downloaded from: http://www.naturalearthdata.com/downloads/10m-raster-data/10m-natural-earth-1/

str_name <- brick("NE1_HR_LC_SR_W.tif")

# Define cropping extent

ext.land <- extent(-140, 0, 55, 84)

# Subset shapefile

shp.land <- crop(str_name, ext.land)

# Plot map with scale, and save to working directory with filename "Map-01.tiff"

dev.off()

tiff("Map-01.tiff", width = 8.75, height = 7, units = 'in', res = 400)

plotRGB(shp.land)

map.scale(-40.0, 57.5, relwidth = 0.15, metric = TRUE, ratio = F)

# Add coordinates from CSV file

points(map.coor$Longitude, map.coor$Latitude, col="black", bg="red", pch=21, cex=1)

dev.off()
